# A Multi‐Structural Radiomic Digital Phenotyping Framework for Deciphering Preclinical Evolution of Degenerative Meniscal Tears

**DOI:** 10.1002/advs.78506

**Published:** 2026-10-30

**Authors:** Shengfa Li, Hao Liu, Yanbin Zhu, Hang Zhang, Qifeng Tao, Wenbo Zhao, Xin Miao, Shanshan Yang, Tianyu Chen, David Hunter, Changhai Ding, Ting Wang, Juan Wang, Wei Chen

**Affiliations:** ^1^ Hebei Medical University Clinical Medicine Postdoctoral Station (Hebei Medical University Third Hospital) Shijiazhuang Hebei China; ^2^ Department of Orthopaedic Surgery NHC Key Laboratory of Intelligent Orthopaedic Equipment Hebei Medical University Third Hospital Shijiazhuang Hebei China; ^3^ Department of Orthopaedics The Third People's Hospital of Chengdu Affiliated Hospital of Southwest Jiaotong University & The Second Affiliated Hospital of Chengdu Chongqing Medical University Chengdu Sichuan China; ^4^ Department of Arthroplasty and Sport Medicine The Third People's Hospital of Chengdu Affiliated Hospital of Southwest Jiaotong University & The Second Affiliated Hospital of Chengdu Chongqing Medical University Chengdu Sichuan China; ^5^ Department of Orthopaedics The Third Affiliated Hospital of Southern Medical University Guangzhou China; ^6^ Clinical Research Centre Zhujiang Hospital Southern Medical University Guangzhou Guangdong China; ^7^ Department of Rheumatology Kolling Institute Royal North Shore Hospital and Sydney Musculoskeletal Health University of Sydney Australia; ^8^ Menzies Institute for Medical Research University of Tasmania Hobart Tasmania Australia; ^9^ Clinical Research Centre Beijing Tsinghua Changgung Hospital Tsinghua Medicine Tsinghua University Beijing China; ^10^ Medical Research Center The Third People's Hospital of Chengdu Affiliated Hospital of Southwest Jiaotong University & The Second Affiliated Hospital of Chengdu Chongqing Medical University Chengdu Sichuan China; ^11^ Joint Surgery Department Hebei Medical University Third Hospital Shijiazhuang Hebei China

**Keywords:** artificial intelligence, digital biomarkers, predictive medicine, tissue remodeling

## Abstract

Degenerative meniscus tears (DMTs) are pivotal in knee osteoarthritis progression, yet conventional MRI often fails to capture the subvisual alterations preceding macroscopic failure. We developed a multi‐structural digital risk stratification framework (MSC‐RM) that integrates standardized radiomic signatures from six knee structures (femur, tibia, cartilages, and menisci) derived from 3D double‐echo steady‐state MRI with baseline clinical risk profiles. Utilizing a longitudinal cohort (*n* = 674), our framework decoded complex degeneration patterns, achieving superior 4‐year predictive performance (AUC 0.871). Survival analysis revealed that high‐risk individuals had a 6.12‐fold greater hazard of developing DMTs than the low‐risk group. Furthermore, a reader test with five resident surgeons demonstrated that MSC‐RM significantly enhanced diagnostic consistency (Kappa increased from 0.38 to 0.55), effectively bridging the expertise gap. By shifting the paradigm from reactive lesion detection to proactive risk management, this framework provides a scalable strategy for precision preventive medicine and establishes digital biomarkers as critical tools for optimizing musculoskeletal health in aging populations.

## Graphical abstract

Subclinical matrix disruption precedes degenerative meniscal tears across the knee joint. A spatial compartmental radiomics model utilizing 3D‐DESS MRI captures early osteochondral destabilization, accurately predicting incident tears up to four years pre‐collapse. Feature attributions uncover coordinated chondral and osseous alterations, advancing non‐invasive risk profiling to guide timely, personalized joint‐preserving interventions.

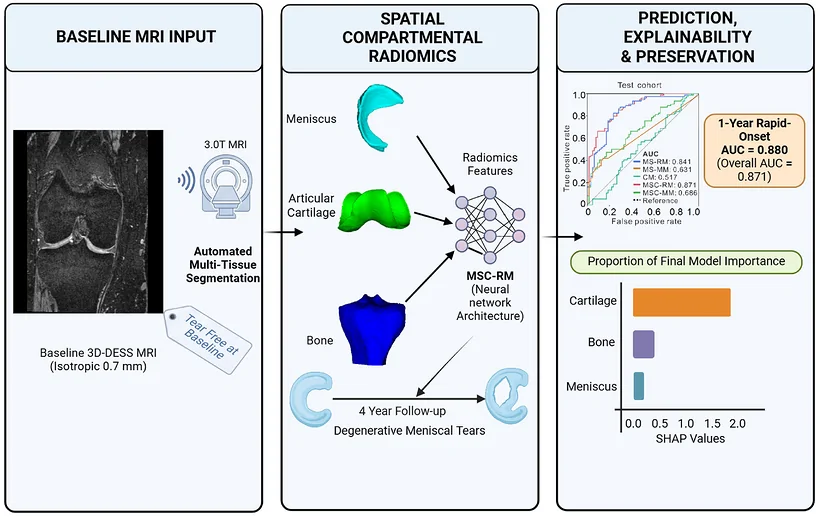

## 1 Introduction

Degenerative meniscus tears (DMTs) represent a critical structural tipping point in the biological continuum of knee joint failure, serving as both a precursor to and a hallmark of osteoarthritis. Unlike acute traumatic injuries, DMTs in aging populations are the culmination of a protracted, multi‐year degenerative process involving complex biochemical and biomechanical remodeling. These tears are inextricably linked to chronic pain, functional impairment, and substantial socioeconomic burdens globally [1]. However, the fundamental scientific challenge remains our inability to decipher the preclinical evolution of these tears before they manifest as irreversible structural damage.

Conventional magnetic resonance imaging (MRI) remains the clinical gold standard for assessing meniscal pathology, yet its current utility is largely reactive rather than proactive. Standard clinical interpretation relies on visual, qualitative descriptions of established lesions, which are inherently insensitive to the subvisual microstructural alterations that precede macroscopic tissue failure [2, 3]. This diagnostic latency creates a critical “blind spot” in musculoskeletal care, where individuals at high risk of future joint collapse remain unidentified until the window for early intervention has closed. Consequently, there is an urgent need for an objective, imaging‐driven framework capable of moving beyond lesion detection toward prospective digital risk stratification.

The emergence of radiomics—the high‐throughput extraction of quantitative data signatures from medical imaging—offers a transformative approach to this problem by treating medical images as minable data repositories rather than mere pictures [4, 5]. While recent advances in deep learning and radiomics have achieved high diagnostic accuracy in identifying existing meniscus pathology [6, 7, 8, 9, 10, 11], these efforts have primarily focused on “snapshot” detection. To date, the potential of MRI‐based digital biomarkers to forecast incident degenerative failure within a longitudinal survival framework remains unexplored. Furthermore, emerging evidence suggests that the meniscus does not degenerate in isolation; rather, it is part of a synergistic “joint‐as‐an‐organ” system where the femur, tibial subchondral bone, and articular cartilage collectively define the biomechanical microenvironment [12, 13, 14, 15, 16]. Capturing this broader context requires a shift from single‐tissue analysis to a multi‐structural holistic approach.

In this study, we developed a Multi‐Structure and Clinical Radiomics Model (MSC‐RM) that integrates high‐dimensional quantitative signatures from six distinct knee anatomical structures—the femur, tibia, femoral and tibial cartilage, and medial and lateral menisci—with baseline clinical risk profiles to predict the four‐year incidence of DMTs. Utilizing high‐resolution isotropic 3D double‐echo steady‐state (3D‐DESS) MRI from a large‐scale longitudinal cohort, we embedded multi‐structure information within a time‐to‐event modeling framework to estimate individualized future risk [17, 18, 19, 20]. Beyond technical validation in an independent cohort, we performed a human‐AI collaboration study to assess how model‐derived probabilistic estimates can bridge the expertise gap among clinicians. By shifting the clinical focus from reactive pathology detection to proactive risk management, this framework establishes a new paradigm for precision preventive medicine in musculoskeletal health.

## 2 Patients and Methods

### 2.1 Study Population and Participants

Participants were selected from the publicly available OAI database (https://nda.nih.gov/oai/, NCT00080171) to construct the Degenerative Meniscus Tears Radiomics (DeMeTeR) cohort (Figure 1). Eligible individuals had no baseline degenerative meniscus tear on MRI according to MOAKS scoring [20]. Incident tears (grades 2–5) identified within four years were defined as case knees, while controls were matched by body mass index (BMI, ±5 kg/m^2^), sex, and age (±5 years) using propensity score matching. A total of 337 case knees and 337 matched controls were included and randomly divided into development and independent test cohorts (8:2 ratio). All participants provided informed consent under institutional review board approval. Data from the OAI cohort included diverse participants, and analyses accounted for sex, race, and age to minimize bias.

**FIGURE 1:**
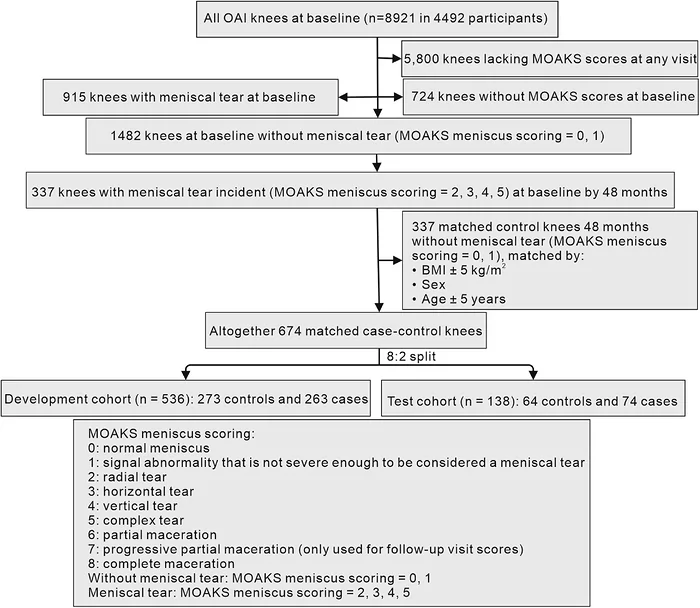
Study flowchart of the DeMeTeR cohort construction and data allocation. Flow diagram illustrating the sequential selection of the nested case‐control cohort from the OAI. Knees lacking MOAKS assessments across all follow‐up time points (*n* = 5,800) and those without valid baseline MOAKS evaluations (*n* = 724) were sequentially excluded. Knees with prior knee surgery (e.g., partial or total meniscectomy) or baseline meniscal maceration (MOAKS scores 6 or 8) were also excluded. A total of 337 knees that developed incident DMTs (MOAKS score 2–5 during any follow‐up year) and 337 matched control knees (no tears across 4 years, MOAKS scores 0–1) were selected from the OAI using a nested case‐control design. Matching was performed based on sex, age (±5 years), and BMI (±5 kg/m^2^). All subjects had no evidence of meniscus tears at baseline (MOAKS score 0–1). The final dataset (*n* = 674 knees) was randomly split into a development cohort (*n* = 536 knees, 263 cases and 273 controls) and an independent test cohort (*n* = 138 knees, 74 cases and 64 controls) in an 8:2 ratio. DeMeTeR: Degenerative Meniscus Tears Radiomics, MOAKS: Magnetic resonance imaging Osteoarthritis Knee Score, OAI: Osteoarthritis Initiative, BMI: Body Mass Index, DMTs: Degenerative Meniscus Tears.

### 2.2 Inclusion and Exclusion Criteria

Participants were included if they had (1) complete baseline 3D‐DESS MRI scans; (2) no DMTs at baseline (MOAKS score 0–1); (3) available clinical data (age, sex, race, BMI, KOOS pain score, medication history); and (4) qualifying longitudinal follow‐up, defined as completing 4 years of annual MRI assessments for controls (to verify tear‐free status through 48 months) or at least 1 year of post‐baseline MRI surveillance for case knees (to permit identification of incident DMTs). Exclusion criteria included (1) prior knee surgery (e.g., partial or total knee replacement, partial or total meniscectomy) or baseline structural disruption, including meniscal maceration (MOAKS score 6–8) and meniscal root tears (MOAKS score 2–5); (2) inflammatory arthritis such as rheumatoid arthritis; (3) lack of qualifying longitudinal clinical or MOAKS follow‐up assessments; and (4) severe MRI motion artifacts or poor image quality unsuitable for image segmentation and radiomic feature extraction.

### 2.3 Clinical Outcome

The primary clinical outcome was the 4‐year incidence of DMTs, evaluated on annual follow‐up MRIs (at 12, 24, 36, and 48 months). The primary clinical outcome was the incidence of DMTs, defined by a Magnetic Resonance Imaging OsteoArthritis Knee Score (MOAKS) meniscus score of 2 (radial tear), 3 (horizontal tear), 4 (vertical tear), or 5 (complex tear) at any follow‐up visit. Two experienced musculoskeletal radiologists independently performed MOAKS evaluations to patient clinical outcomes, demonstrating substantial to excellent inter‐reader agreement (κ = 0.61–1.0). Time to meniscus tear was calculated from baseline MRI to the first detection of a qualifying MOAKS score. To account for differential follow‐up durations and event timing, Kaplan–Meier survival curves and Cox proportional hazards regression were employed. Knees experiencing an incident DMTs were assigned their documented event time (12, 24, 36, or 48 months), whereas event‐free knees were right‐censored at the 48‐month follow‐up.

### 2.4 MRI Semiquantitative MOAKS Assessment

Beyond meniscal tear grading, whole‐organ semiquantitative assessment was performed using the standardized MOAKS system across multiple joint compartments to build comparator benchmark models (Table S4). Specifically, this included: (1) meniscal morphology, extrusion, signal abnormality, and root tears for both medial and lateral menisci; (2) articular cartilage morphology across 12 subregions of the femur and tibia; and (3) subchondral bone marrow lesions (BMLs, quantified by lesion extent and percentage edema) and marginal osteophytes across corresponding subregions of the femur and tibia.

### 2.5 MRI Acquisition and Segmentation

All participants underwent 3D‐DESS knee MRI using a 3.0 T scanner (Magnetom Trio, Siemens Healthcare, Erlangen, Germany) following the OAI imaging protocol (Table S1) (slice thickness = 0.7 mm, field of view = 140 mm, TR = 16.3 ms, TE = 4.7 ms) [21]. Six anatomical structures were analyzed: femur, femoral cartilage, tibia, tibial cartilage, medial meniscus, and lateral meniscus (Figure S1).

Automated segmentation of these structures [22] was performed using convolutional neural network (CNN)‐based methods [23, 24] to define volumes of interest (VOIs), which were subsequently reviewed and manually adjusted by two independent musculoskeletal researchers (with 5 and 7 years of experience in musculoskeletal image processing and orthopedics). Both researchers were strictly blinded to all longitudinal outcomes, including DMTs incidence, follow‐up MRI findings, time‐to‐event data, and participant clinical scores. Segmentation quality was evaluated by Dice Similarity Coefficients (DSC) in a random subset of 20 knees (Table S2). Automated CNN segmentations showed good‐to‐excellent agreement with expert adjustments (DSC > 0.80 across all structures, Table S2), and inter‐reader agreement between the two manual adjustments was high (DSC = 0.87–0.91, Table S2). Following this technical validation, automated CNN segmentations were deployed across the entire cohort without manual intervention, and all downstream radiomic features were extracted directly from these automated VOIs to ensure an objective, fully scalable, and operator‐independent processing pipeline. All MRI reviews and manual refinements were performed using itk‐SNAP v3.8.0 (www.itksnap.org).

### 2.6 Signal Feature Map of Load‐Bearing Tissues

We additionally developed feature maps of multi‐structure based on their 3D‐DESS signal intensity (mean voxel intensity value), building upon previous studies that visualized knee MRI texture patterns [25]. Three‐dimensional spatial feature maps were rendered in 3D Slicer (version 4.11) by projecting the segmented VOIs onto the co‐registered 3D‐DESS volumes. Pseudo coloring was implemented using the Rainbow Lookup Table (LUT) to display signal intensity gradients. The localized numerical annotations displayed on the feature maps—designated as mean voxel intensity values (derived via the Label Text → Value module)—quantified the average signal intensity within each load‐bearing subregion, allowing direct comparison of early degenerative changes between knees with and without incident DMTs.

### 2.7 Radiomics Feature Extraction

Radiomics features were extracted from the segmented 3D‐DESS images using the open‐source Standardized Environment for Radiomics Analysis (SERA) package (https://visera.ca/) [26]. SERA extracts radiomic features based on the Image Biomarker Standardization Initiative (IBSI) guidelines [27], ensuring reproducibility and standardization across studies. Extracted features included shape descriptors, first‐order statistics, and texture features derived from gray‐level co‐occurrence matrix (GLCM), gray‐level size zone matrix (GLSZM), and gray‐level run‐length matrix (GLRLM). These texture features capture spatial relationships, size variation, and directional patterns of gray‐level intensities within the regions of interest. Feature robustness was ensured by intra‐ and inter‐observer reproducibility analysis.

To ensure feature robustness, intra‐ and inter‐observer reproducibility analyses were performed on a random subset of 20 baseline knees using intraclass correlation coefficients (ICC; two‐way random‐effects model, absolute agreement). One reader repeated the analysis after a 4‐week interval to evaluate intra‐observer stability. All radiomic features in the radiomics models exhibited high stability (ICC = 0.81–0.96; Table S4).

### 2.8 Clinical Variables and Feature Selection

Baseline clinical variables included race, sex, BMI, glucosamine use, Physical Activity Scale for the Elderly (PASE) score, and KOOS subscales (pain, symptoms, quality of life). A total of 2,947 radiomic and clinical features were extracted. To ensure feature selection stability, least absolute shrinkage and selection operator (LASSO) penalized regression was implemented with 10‐fold cross‐validation across 1,000 resamplings (maximum convergence iterations = 10 000). Features with non‐zero coefficients across resamplings were retained (Figures S2 and S3). Forty‐eight non‐zero features (40 radiomic, 8 clinical) were retained and used to build predictive models via a shallow neural network, with regularization and AICc minimization in SAS 9.4 to ensure model parsimony.

The selected features were used to develop the MSC‐RM and six single‐structure radiomics models (Femur Radiomics Model (FE‐RM), Femoral Cartilage Radiomics Model (FC‐RM), Tibia Radiomics Model (TI‐RM), Tibial Cartilage Radiomics Model (TC‐RM), Lateral Meniscal Radiomics Model (LM‐RM), Medial Meniscal Radiomics Model (MM‐RM)) and one integrated Multi‐Structure Radiomics Model (MS‐RM) were developed. For clinical and semiquantitative benchmarking, six corresponding single‐structure MOAKS models (Femur MOAKS Model (FE‐MOM), Femoral Cartilage MOAKS Model (FC‐MOM), Tibia MOAKS Model (TI‐MOM), Tibial Cartilage MOAKS Model (TC‐MOM), Lateral Meniscal MOAKS Model (LM‐MOM), Medial Meniscal MOAKS Model (MM‐MOM)), an integrated Multi‐Structure MOAKS Model (MS‐MOM), and a Clinical Model (CM) based strictly on the eight baseline clinical variables were established. Ultimately, our primary proposed framework—the MSC‐RM—was established by fusing 40 robust radiomic features across all six compartments with the eight clinical variables, matched against corresponding Multi‐Structure and Clinical MOAKS Model (MSC‐MOM) which incorporated all six compartments MOAKS scores and clinical variables. These comparator models enabled assessment of the incremental predictive value of radiomics over traditional semiquantitative scoring and clinical data alone.

### 2.9 Model Development and Validation

The DeMeTeR cohort included 674 knee MRIs from 660 participants, randomly divided into a development cohort (*n* = 536; 263 cases, 273 controls) and an independent test cohort (*n* = 138; 74 cases, 64 controls) at an 8:2 ratio (Figure 2). To promote model generalizability across broad population variability and prevent overfitting to pairwise dyadic constraints, allocation was conducted at the participant level without binding matched pairs to the same partition. All feature standardization, selection, and model training routines were executed strictly within the development/test cohort to ensure complete independence of the test set.

**FIGURE 2:**
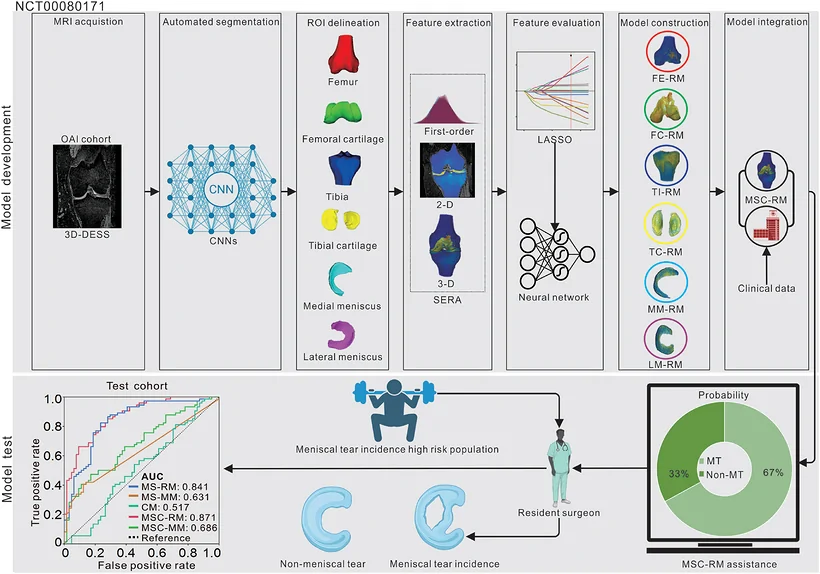
Workflow of radiomics‐based predictive model development for DeMeTeR cohort. The analysis pipeline consisted of five major steps: (1) baseline 3D‐DESS MRI acquisition and segmentation of six anatomical structures (femur, femoral cartilage, tibia, tibial cartilage, medial meniscus, and lateral meniscus) using CNN‐based auto‐segmentation and manual correction; (2) radiomics feature extraction from each VOI using the SERA package, following IBSI standards; (3) clinical data collection at baseline, including demographics, KOOS subscales, and relevant risk factors; (4) feature selection using LASSO logistic regression repeated 1,000,000 times; and (5) model construction using a shallow neural network to develop structure‐specific models (e.g., FE‐RM, FC‐RM, TI‐RM, TC‐RM, LM‐RM, and MM‐RM) and a final integrated MSC‐RM. Model performance was evaluated in a separate test cohort. DeMeTeR: Degenerative Meniscus Tears Radiomics, CNN: Convolutional Neural Networks, VOI: Volume Of Interest, SERA: Standardized Environment for Radiomics Analysis, IBSI: Image Biomarker Standardization Initiative, KOOS: Knee injury and Osteoarthritis Outcome Score, LASSO: Least Absolute Shrinkage and Selection Operator, FE‐RM: Femur Radiomics Model, FC‐RM: Femoral Cartilage Radiomics Model, TI‐RM: Tibia Radiomics Model, TC‐RM: Tibial Cartilage Radiomics Model, LM‐RM: Lateral Meniscus Radiomics Model, MM‐RM: Medial Meniscus Radiomics Model, MSC‐RM: Multi‐Structure and Clinical Radiomics Model.

A predictive model was constructed using a shallow neural network. The network comprised one hidden layer with a hyperbolic tangent (TanH) activation function and was trained exclusively in the development cohort [28]. Model performance was evaluated in the test cohort using Cohen's kappa, area under the ROC curve (AUC), sensitivity, and specificity with 95% confidence intervals. Participants were stratified into high‐ and low‐risk groups based on the optimal Youden index cutoff. Kaplan‐Meier survival and log‐rank tests compared time‐to‐event outcomes, and Cox regression estimated hazard ratios with 95% CIs. All computations were performed on a standard workstation (Core i5, 16 GB RAM).

### 2.10 MRI Reader Test

To assess the potential clinical utility of the MSC‐RM model, five orthopedic resident surgeons independently evaluated the risk of meniscus tear for each subject in the test cohort, first without and then with the assistance of the MSC‐RM model output. Changes in diagnostic sensitivity and specificity were recorded and compared.

### 2.11 Model Interpretability and Feature Attribution

To provide post‐hoc explainability and determine the biological drivers of the final neural network model (MSC‐RM), feature attribution was performed using SHapley Additive exPlanations (SHAP) based on cooperative game theory. Kernel SHAP was implemented to quantify the marginal contribution of each feature to the model's log‐odds output across the independent test cohort (*n* = 138). The median feature vector of the development cohort (*n* = 536) served as the baseline reference. Global feature importance was computed as the mean absolute SHAP value (mean |SHAP|) for each predictor. To quantify structural contributions across the joint, predictors were mapped into six anatomical load‐bearing compartments—medial meniscus, lateral meniscus, tibial cartilage, femoral cartilage, femur, and tibia—along with clinical covariates, and their relative percentage contributions were aggregated. Local and global feature distributions were visualized using SHAP beeswarm plots and compartmental importance bar charts.

### 2.12 Clinical Utility and Decision Curve Analysis

To formally assess the clinical translatability and practical net benefit of the predictive models across varied clinical decision thresholds, Decision Curve Analysis (DCA) was conducted in the test cohort (*n*  =  138) following the methodology formulated by Vickers and Elkin [29]. Standardized net benefit was computed by balancing the proportion of true positives against the proportion of false positives weighted by the relative harm of overtreatment across threshold probabilities (*p_t_*) ranging from 0.01 to 0.85:

$$\mathrm{Net}\;\mathrm{Benefit}\;=\frac{\mathrm{True}\;\mathrm{Positives}}{N}\;-\frac{\mathrm{False}\;\mathrm{Positives}}{N}\times \left(\frac{p_{t}}{1-p_{t}}\right)$$

The final multivariable model (MSC‐RM) was comparatively evaluated against the CM and two default clinical decision paradigms: the “treat‐all” strategy (assuming all patients are at high risk and receive preventative interventions) and the “treat‐none” strategy (assuming no patients receive intervention, with a net benefit of zero).

### 2.13 Cumulative Time‐Horizon Dynamic AUC and Subgroup Analysis

To evaluate the longitudinal temporal stability and discrimination of the models across varying follow‐up windows, cumulative time‐horizon AUC analyses were performed in the test cohort. Incident cases were stratified by the documented annual time‐to‐tear onset (tearyear∈{1,2,3,4}). Cumulative dynamic AUCs along with 95% CIs (derived via 1,000 bootstrap iterations) were computed for rapid onset (≤1 year, *n* = 50; ≤2 years, *n* = 58) and extended horizons (≤3 years, *n* = 64; ≤4 years, *n* = 74) against control knees (*n* = 64) without tear manifestation across 4 years.

### 2.14 Missing Data Handling

All radiomic features had complete data. Clinical candidate predictors exhibited minimal missingness (< 0.5% per variable; race, *n* = 3; baseline glucosamine use, *n* = 2; PASE score, *n* = 2; KOOS quality of life, *n* = 1). Missing categorical and continuous values were imputed using the mode and median derived exclusively from the development cohort to prevent data leakage.

### 2.15 Statistical Analysis

Baseline characteristics were compared using unpaired *t*‐tests or Mann–Whitney *U*‐tests for continuous variables and chi‐squared tests for categorical variables. The longitudinal associations between individual structural features/scores and incident DMTs were analyzed using Generalized Estimating Equations (GEE) adjusted for age, sex, BMI, knee side, race, and KOOS pain score [14], with an autoregressive correlation structure to account for repeated measures. For 4‐year time‐to‐event outcomes, cumulative incidence was visualized using Kaplan–Meier curves and compared via log‐rank tests. An unadjusted Cox proportional hazards model was utilized to evaluate risk stratification by the final model (MSC‐RM), because the model already incorporates baseline clinical variables, making further adjustment redundant and prone to collinearity. Feature selection (LASSO), neural network construction, GEE modeling, and DeLong tests were conducted in SAS 9.4. Radiomic feature extraction and Dice Similarity Coefficient (DSC) calculations were performed in MATLAB R2021a, while ROC analysis and time‐to‐event evaluations (Kaplan‐Meier and Cox regression) were conducted in R 4.1.1 using the pROC and survival packages. The Youden index determined the optimal cutoff for risk stratification. Two‐sided *p* < 0.05 was considered statistically significant.

## 3 Results

### 3.1 Study Population and Baseline Characteristics

A total of 674 knees from 660 participants were included in the DeMeTeR cohort, with 536 knees (263 cases, 273 controls) assigned to the development cohort and 138 knees (74 cases, 64 controls) to the test cohort. Baseline characteristics are summarized in Table 1 and Table S3. In the development cohort, no significant differences were found between cases and controls in age, sex, BMI, prior knee injury or surgery, NSAID use, or KOOS scores. In the test cohort, cases were slightly older (61.5 ± 8.1 vs. 56.4 ± 8.7 years, *p* < 0.001) and had lower KOOS symptom scores (*p* = 0.006). Overall, both cohorts were well balanced, with only NSAID use differing modestly between datasets (8.8% vs. 3.6%, *p* = 0.043).

**TABLE 1:** Baseline characteristics of case and control groups in the development cohort and test cohort.

	Development cohort (*n* = 536)			Test cohort (*n* = 138)		
	Control (*n* = 273)	Case (*n* = 263)	*p‐*value	Control (*n* = 64)	Case (*n* = 74)	*p‐*value
Age (year)^a^	61.0 ± 9.2	60.6 ± 8.7	.636	56.4 ± 8.7	61.5 ± 8.1	< .001
Female^b^	148 (54%)	135 (51%)	.505	35 (55%)	48 (65%)	.225
BMI (kg/m^2^)^a^	28.1 ± 4.8	28.4 ± 4.9	.473	28.0 ± 4.6	27.7 ± 4.3	.714
Knee injury^b^	72 (27%)	68 (26%)	.812	19 (30%)	21 (28%)	.820
Knee surgery^b^	36 (13%)	22 (8%)	.078	10 (16%)	7 (9%)	.274
NSAIDs use^b^	20 (7%)	27 (10%)	.224	2 (3%)	3 (4%)	.772
Pain medicine use^b^	71 (26%)	78 (30%)	.374	17 (27%)	15 (20%)	.384
KOOS pain^c^	92 (78, 100)	94 (83, 100)	.214	90 (78, 100)	89 (81, 100)	.606
KOOS symptom^c^	93 (82, 100)	93 (86, 100)	.36	93 (82, 100)	93 (82, 100)	.006
KOOS funtion, sports, and recreational activities^c^	83 (60, 100)	83 (67, 95)	.772	83 (65, 100)	81 (56, 100)	.525
KOOS quality of life^c^	69 (56, 88)	69 (56, 88)	.670	75 (56, 88)	69 (56, 88)	.958

Data are mean ± SD, number (%), or median (IQR).

^a^ Unpaired *t*‐tests are used for differences between means.

^b^ χ2 tests are used for differences between proportions.

^c^ Mann–Whitney tests are used for differences between ranks.

BMI: Body Mass Index, NSAIDs: Non‐Steroidal Anti‐Inflammatory Drugs, KOOS: Knee Outcomes in Osteoarthritis Survey, SD: Standard Deviation, IQR: Interquartile Range.

### 3.2 Radiomics Feature Extraction and Selection

The DSCs calculated for validating segmentation reliability exceeded 0.80 for all structures (Table S2). The final MSC‐RM retained 48 non‐zero features: 40 were radiomic features and 8 were clinical variables (Table S4, Figure S3). The selected features spanned all six structures and captured a wide range of imaging biomarkers including first‐order intensity statistics, morphological attributes, and advanced texture descriptors (e.g., GLCM, GLSZM, GLRLM).

### 3.3 Model Performance and Validation

To visualize localized tissue alterations across the joint compartments, regional 3D signal intensity maps were generated. The displayed quantitative metrics (mean voxel intensity values) demonstrated distinct signal variations within the load‐bearing VOIs between progressors and non‐progressors. As a representative case illustration, longitudinal analysis of the medial meniscus (Figure 3) showed persistently elevated mean voxel intensity values in the case knee across all time points, preceding a degenerative tear at the 4‐year follow‐up, indicating that radiomic alterations may occur years before morphological failure. In the representative case knee compared with the control knee, signal intensity patterns across load‐bearing knee structures were visualized using baseline 3D‐DESS MRI feature maps (Figure 4a–f, Figure S4). Signal intensity in the femur, tibia, cartilage, and both menisci was consistently higher in case knees than in controls, suggesting that subtle subvisual changes precede structural damage.

**FIGURE 3:**
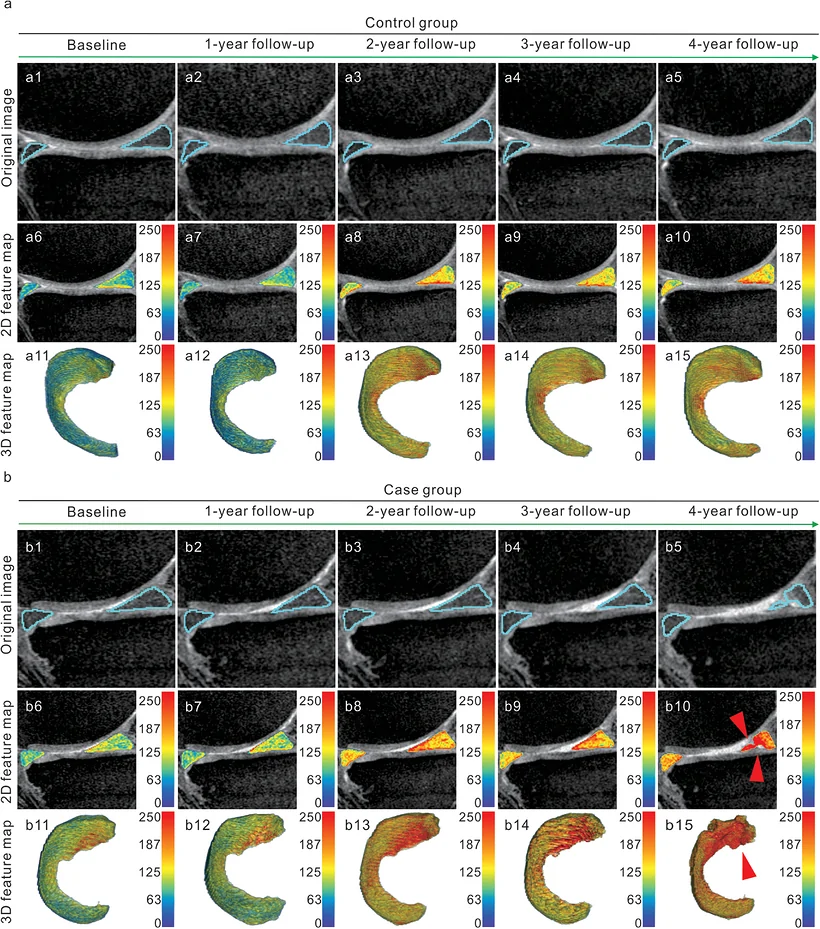
Longitudinal changes in medial meniscus features between case and control knees from baseline to 4‐year follow‐up. Representative examples of the medial meniscus from a case knee (b) and a control knee (a) are shown at baseline, 1‐, 2‐, 3‐, and 4‐year follow‐up. Each time point includes the original MRI image, 2D feature map, and 3D feature map. The case knee exhibited consistently higher medial meniscus mean voxel intensity values compared with the control knee across all five time points, preceding the incidence of a degenerative meniscus tear at the 4‐year follow‐up (red arrow). This figure illustrates the progressive radiomic alterations in the medial meniscus before structural failure, supporting the model's ability to capture early subvisual tissue changes. Representative 3D signal feature maps generated in 3D Slicer using a Rainbow lookup table. Color gradients illustrate the spatial distribution of signal intensity, where warmer colors correspond to higher mean voxel intensity values within the segmented articular volumes of interest. 2D: 2D, 3D: 3D, MRI: Magnetic Resonance Imaging.

**FIGURE 4:**
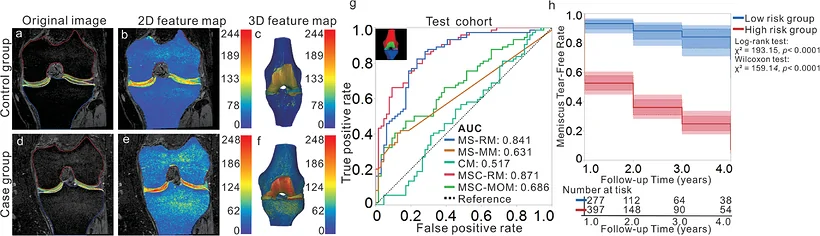
Model performance and visual analysis of degenerative meniscus tear prediction. Baseline 3D‐DESS MRI signal feature maps across load‐bearing knee structures in representative matched knees that developed DMTs (case knee, d–f) versus those that did not (control knee, a–c). Pseudo colored feature maps were generated in 3D Slicer using the Rainbow Lookup Table, where warmer hues correspond to higher mean voxel intensity values within the segmented volumes of interest. In this representative illustration, elevated signal intensity patterns are visually apparent across the load‐bearing compartments in the case knee, reflecting early subclinical tissue alterations prior to macroscopic tear manifestation. (g) ROC curves of the MSC‐RM model in the test cohort, showing an AUC of 0.871 (95% CI: 0.802‐0.918), with optimal cutoff determined by Youden index = 0.434 (sensitivity = 71.9%, specificity = 83.8%). (h) Kaplan‐Meier survival curves showing cumulative incidence of DMTs in high‐risk versus low‐risk groups as defined by MSC‐RM predictions. A significant separation was observed between the two curves (log‐rank *p* < .0001), and the high‐risk group had a 6.12‐fold higher hazard of developing tears (HR = 6.12, 95% CI: 4.31‐8.68). 3D‐DESS: 3D Double‐Echo Steady‐State, MRI: Magnetic Resonance Imaging, ROC: Receiver Operating Characteristic, MSC‐RM: Multi‐Structure and Clinical Radiomics Model, AUC: Area Under the receiver operating characteristic Curve, CI: Confidence Intervals, DMTs: Degenerative Meniscus Tears.

At the overall cohort level (*n* = 674), quantitative radiomic profiling confirmed marked microstructural divergence between incident DMTs knees and matched controls (Table S10). Specifically, case knees exhibited significantly elevated mean voxel intensity in the medial meniscus compared with controls (ih_mean: 53.8 ± 3.8 vs. 52.4 ± 4.5, *p* < 0.001). Furthermore, higher‐order textural disorganization was notably increased in case knees, as reflected by significantly higher 3D joint entropy in both the medial meniscus (9.6 ± 0.3 vs. 9.4 ± 0.4, *p* < 0.001) and tibial cartilage (9.6 ± 0.4 vs. 9.4 ± 0.5, *p* < 0.001).

In the independent test cohort (*n* = 138), the MSC‐RM achieved the best predictive performance for incident DMTs (AUC = 0.871, 95% CI: 0.802‐0.918; sensitivity = 71.9%; specificity = 83.8%; Youden index = 0.614; cutoff = 0.434) (Table 2, Figure 4g). Beyond the primary Youden‐derived threshold of 0.434, shifting the cutoff to a conservative screening setting further suppressed false positives to achieve 98.4% specificity and a positive predictive value of 97.6% while preserving 55.4% sensitivity in the test cohort (Table S12). The MS‐RM without clinical variables also performed well (AUC = 0.841), whereas the CM showed limited discrimination (AUC = 0.517). The MOAKS‐based model (MSC‐MOM) achieved moderate performance (AUC = 0.686) but was significantly inferior to MSC‐RM (*p* < 0.001, DeLong test).

**TABLE 2:** Prediction performance of models in the development and test cohort.

Model name	Development cohort (*n* = 536)	Test cohort (*n* = 138)
MS‐RM		
Sensitivity	81.7% (88/263)	77.0% (88/74)
Specificity	81.7% (74/273)	78.1% (74/64)
AUC (95% CI)	0.895 (0.866‐0.918)	0.841 (0.761‐0.898)
MS‐MOM		
Sensitivity	51.7% (136/263)	40.5% (30/74)
Specificity	91.6% (250/273)	85.9% (55/64)
AUC (95% CI)	0.735 (0.700–0.768)	0.631 (0.553–0.702)
Clinical model		
Sensitivity	88.2% (232/263)	87.8% (65/74)
Specificity	19.4% (53/273)	15.6% (10/64)
AUC (95% CI)	0.565 (0.516‐0.613)	0.517 (0.419–0.614)
MSC‐RM		
Sensitivity	78.8% (215/263)	71.9% (46/74)
Specificity	82.9% (218/273)	83.8% (62/64)
AUC (95% CI)	0.890 (0.859–0.914)	0.871 (0.802–0.918)
MSC‐MOM		
Sensitivity	55.5% (146/263)	43.2% (32/74)
Specificity	84.6% (231/273)	84.4% (54/64)
AUC (95% CI)	0.777 (0.736–0.813)	0.686 (0.593–0.767)

Data are percentages (numerator/denominator for percentages) or mean (95% CI).

AUC: Area Under the receiver operating characteristic Curve, CI: Confidence Interval, MS‐RM: Multi‐Sructure Radiomic Model, MS‐MOM: Multi‐Sructure MOAKS Model, MSC‐RM: Multi‐Sructure plus Clinical variable Radiomic Model, MSC‐MOM: Multi‐Sructure plus Clinical variable MOAKS Model, MOAKS: Magnetic resonance imaging OsteoArthritis Knee Score, sensitivity = TP/(TP+FN), specificity = TN/(TN+FP), TP: True Positive, TN: True Negitive, FP: False Positive, FN: False Negitive.

Among single‐structure models, the medial meniscus radiomics model performed best (AUC = 0.750), followed by tibial cartilage (AUC = 0.713) and femur (AUC = 0.717) (Table S5, Figure S5). Pairwise AUC comparisons confirmed that MSC‐RM outperformed all radiomics, MOAKS‐based, and CM (all *p* < 0.001). Confusion matrices (Table S6, Figure S6) demonstrated the best balance of sensitivity and specificity for MSC‐RM (κ = 0.560), supporting its clinical utility for early identification of high‐risk individuals.

### 3.4 Time‐to‐Event Analysis

Time‐to‐event analysis confirmed the prognostic value of the MSC‐RM model. Using the optimal cutoff (0.434), the test cohort was divided into high‐risk (*n* = 397) and low‐risk (*n* = 277) groups. Kaplan–Meier curves showed a marked separation in cumulative incidence of DMTs over four years (log‐rank χ^2^ = 193.15, *p* < .0001) (Figure 4h). Cox regression revealed that high‐risk participants had a 6.12‐fold greater hazard of developing tears than low‐risk participants (HR = 6.12, 95% CI: 4.31–8.68, *p* < .0001). These results demonstrate the model's strong capacity for longitudinal risk stratification and its potential clinical utility for dynamic, time‐based prediction.

### 3.5 GEE Analysis

A GEE model was used to assess the association between MSC‐RM–predicted risk and degenerative meniscus tear incidence while adjusting for age, sex, BMI, knee side, race, and KOOS pain score (Table S7). The high‐risk group showed significantly increased odds of developing degenerative tears (adjusted OR = 6.22, 95% CI: 4.34–8.91, *p* < .0001), consistent with the hazard ratio observed in time‐to‐event analysis, confirming the model's independent predictive value beyond clinical confounders.

### 3.6 MRI Reader Test

To assess the clinical utility of the MSC‐RM, a reader test was conducted with five orthopedic resident surgeons (2–3 years of experience in sports medicine). Each reader evaluated baseline 3D‐DESS MRI and clinical data to predict incident DMTs, both with and without access to MSC‐RM outputs (Figure 5a). The model provided individualized tear probabilities via a shallow neural network, color‐coded by risk level (red: high‐risk >0.434; black: low‐risk ≤0.434) for easy interpretation (Figure 5b).

**FIGURE 5:**
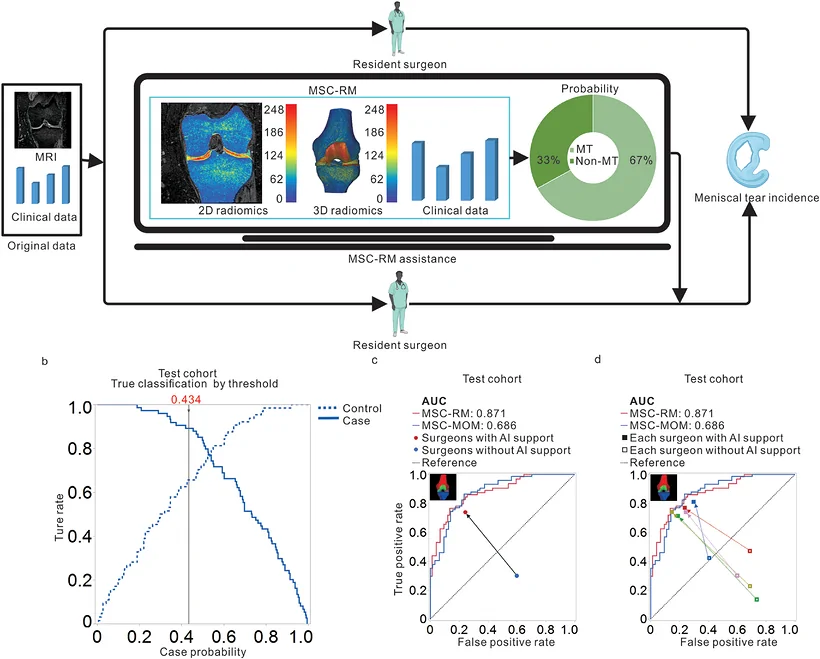
Clinical impact of MSC‐RM model on orthopedic resident diagnostic performance. (a) Illustration of the reader study design: five orthopedic resident surgeons (each with 2–3 years of arthroplasty sports medicine experience) independently assessed baseline 3D‐DESS MRI scans and clinical variables for degenerative meniscus tear risk prediction under two conditions, without (blinded) and with access to MSC‐RM output. Model predictions were displayed using color‐coded probabilities, with red font indicating high‐risk classification (≥0.434) and black font for low‐risk. (b) Example of resident‐facing MSC‐RM risk display showing predicted probability and threshold classification. (c) Comparison of diagnostic sensitivity and specificity across five readers before and after MSC‐RM assistance. Model‐guided interpretation significantly improved average sensitivity from 63.1% to 74.2%, and specificity from 54.7% to 80.0%. (d) Inter‐reader agreement improved with model assistance, as reflected by an increase in mean Cohen's kappa from 0.38 to 0.55. These results highlight the utility of MSC‐RM as a clinical decision support tool for improving diagnostic consistency and accuracy in early‐career orthopedic surgeons. 3D‐DESS: 3D Double‐Echo Steady‐State, MRI: Magnetic Resonance Imaging, MSC‐RM: Multi‐Structure and Clinical Radiomics Model.

Without model assistance, the mean diagnostic sensitivity and specificity were 63.1% and 54.7%, respectively. With MSC‐RM support, these improved to 74.2% and 80.0% (Tables S8 and S9, Figure 5c,d). Inter‐reader agreement also increased substantially, with the mean Cohen's kappa rising from 0.38 to 0.55, indicating enhanced diagnostic consistency.

### 3.7 Feature Attribution and Compartmental Drivers of DMTs Prediction

Post‐hoc Kernel SHAP analysis on the independent test cohort (*n* = 138) revealed the hierarchical contributions of specific radiomic signatures and anatomical compartments driving high‐risk predictions (Figure S7). Among the top 20 predictors ranked by global importance (mean |SHAP|), features capturing microstructural disorganization within articular cartilage and menisci dominated the model output (Figure S7a, b). Specifically, elevated baseline textural heterogeneity (e.g., dzm_ldlge_2d_fc, cm_joint_max_3d_comb_fc, and cm_diff_avg_2d_comb_tc) was positively associated with an increased predicted risk of incident DMTs (Figure S7a).

In terms of compartmental attribution, meniscal tissues constituted the largest primary predictive driver, contributing 43.0% of total model importance (medial meniscus: 21.5%, lateral meniscus: 21.5%) (Figure S7c). Articular cartilage compartments provided the second‐largest contribution at 31.6% (tibial cartilage: 17.5%, femoral cartilage: 14.1%), followed by bone structures at 17.2% (femur: 13.9%, tibia: 3.3%) and clinical covariates at 8.2% (Figure S7c).

### 3.8 Clinical Utility and Decision Curve Analysis

Beyond prospective discrimination, DCA in the independent test cohort (*n* = 138) confirmed the clinical utility of the MSC‐RM model (Figure S8). Across a broad spectrum of clinically realistic threshold probabilities (*p_t_* between 0.05 and 0.85), MSC‐RM conferred a substantially higher net clinical benefit than either the “treat‐all” or “treat‐none” default strategies. Furthermore, the net benefit of MSC‐RM consistently outperformed that of the conventional clinical model across all decision thresholds. For example, at a threshold probability of 30%, the net benefit of MSC‐RM was 0.415 compared to 0.337 for both the CM and the “treat‐all” strategy. Even at higher threshold probabilities (*p_t_* > 0.05), where the clinical model's net benefit deteriorated rapidly toward baseline, MSC‐RM retained sustained clinical utility, confirming that multi‐compartmental radiomics features provide actionable additive value beyond conventional clinical risk factors.

### 3.9 Longitudinal Predictive Trajectory and Time‐Horizon Dynamics

Time‐horizon dynamic evaluation in the test cohort demonstrated remarkable discriminative stability across all follow‐up intervals (Figure S9). For rapid‐onset DMTs developing within the first year (≤1 year, *n* = 50), the MSC‐RM model achieved its peak predictive performance with an AUC of 0.880 (95% CI: 0.814‐0.938), markedly outperforming the clinical model (AUC = 0.466, 95% CI: 0.35‐0.581). Predictive accuracy was sustained across subsequent cumulative horizons, yielding an AUC of 0.867 (95% CI: 0.801‐0.925) for ≤2 years (*n* = 58), 0.868 (95% CI: 0.803‐0.924) for ≤3 years (*n* = 64), and 0.871 (95% CI: 0.802‐0.918) for the full 4‐year period (*n* = 74) (Figure S9a, Table S11). Baseline predicted probabilities generated by MSC‐RM exhibited decisive risk stratification: knees developing tears within 1 year exhibited a median risk probability of 0.768 (mean: 0.730 ± 0.216), whereas asymptomatic controls without incident tears throughout 4 years remained clustered at a low baseline risk (median: 0.308; mean: 0.339 ± 0.208) (Figure S9b).

## 4 Discussion

In this study, we present a multi‐structural digital framework for the prospective risk stratification of DMTs, shifting the paradigm from reactive lesion detection to proactive risk management. By integrating high‐dimensional radiomic signatures from six distinct knee structures within a time‐to‐event modeling paradigm, our framework demonstrates that the preclinical evolution of joint failure can be decoded from baseline MRI long before macroscopic structural breakdown. The robust performance observed in our independent cohort underscores the potential of digital phenotyping to provide individualized prognostic insights that surpass the resolution of conventional semi‐quantitative scoring and clinical assessments.

Most existing artificial intelligence applications in musculoskeletal imaging have focused on the cross‐sectional identification of established pathology [30]. While these “snapshot” models achieve high diagnostic accuracy, they often treat the meniscus in isolation and utilize complex “black‐box” architectures that offer limited biological interpretability [11, 31]. In contrast, our framework adopts a “joint‐as‐an‐organ” perspective, recognizing that DMTs are not focal events but manifestations of a systemic failure within the joint microenvironment. By extending feature extraction to the interconnected osseous and load‐bearing articular structures—including subchondral bone and articular cartilage—our model captures the synergistic biomechanical and biochemical alterations that define the pre‐tear state. This holistic approach aligns with the evolving understanding of joint degeneration as a spatially coordinated process across multiple tissues.

A critical finding of our work is that radiomic signatures can serve as early digital biomarkers, capturing subvisual tissue remodeling—such as subtle changes in collagen network organization or subchondral bone mineral density—that precedes the visual threshold of human experts. While prior work has identified prognostic signals within the meniscus itself [6], our multi‐structural integration provides a more comprehensive representation of the load‐bearing microenvironment. The high‐resolution, isotropic 3D‐DESS sequence played a pivotal role here, allowing the extraction of robust texture and wavelet features that reflect the internal heterogeneity of the tissues [21, 27]. By embedding these signals within a survival analysis framework, we move beyond binary classification toward probabilistic risk trajectories, providing a quantitative foundation for longitudinal musculoskeletal care.

The clinical implications of this framework extend to the concept of precision preventive medicine. Identifying individuals at an elevated risk (with a 6.12‐fold higher likelihood of DMTs) creates a critical window for intervention, such as neuromuscular training or load modification, before advanced structural damage and subsequent osteoarthritis become inevitable [32]. Furthermore, our reader test results—demonstrating an increase in diagnostic consistency (Kappa 0.38 to 0.55)—illustrate a feasible pathway for Human‐AI collaboration. Rather than replacing clinical judgment, this digital framework acts as an “expert‐level” decision support tool, bridging the expertise gap and standardizing risk communication across varying levels of clinical experience.

Beyond achieving robust prospective discrimination, deciphering the model's decision architecture via SHAP attribution provides vital insights into the pathophysiological sequence preceding degenerative tears. Reviewing the relative contributions of bone versus cartilage and meniscal tissues confirmed that while localized meniscal fibrocartilage deterioration (accounting for 43.0% of total importance) directly dictates tear occurrence, adjacent femoral and tibial hyaline cartilage (31.6%) and bone remodeling (17.2%) serve as critical co‐drivers.

Biomechanically, the prominence of tibial and femoral cartilage texture entropy (cm_diff_avg_2d_comb_tc, dzm_ldlge_2d_fc) underscores that subtle cartilage surface shear stress alteration is intimately coupled with abnormal meniscal hoop stress transmission. Simultaneously, the non‐negligible attribution of femoral and tibial bone features (ngl_hde_2_5d_fe, cm_diff_entr_2_5d_avg_ti) supports the whole‐organ joint degradation paradigm: early bone trabecular stress alterations parallel or precede macroscopic meniscal structural failure. By translating black‐box neural network outputs into anatomical and feature‐level contributions, these findings furnish clinicians with intuitive imaging markers to assess pre‐structural meniscal vulnerability across the osteochondral unit.

Beyond standard discrimination metrics like the C‐statistic, demonstrating tangible clinical net benefit is crucial for integrating predictive algorithms into orthopedic practice. DCA in the independent test cohort confirmed that utilizing MSC‐RM confers a superior net clinical benefit over conventional clinical assessment and default “treat‐all” or “treat‐none” strategies across a wide threshold range of 5%–85%. In clinical settings, deciding whether to initiate proactive joint‐preservation interventions—such as targeted neuromuscular training, load‐modifying braces, or intensive activity modification—requires balancing prophylactic efficacy against the burdens of over‐intervention [33]. By minimizing false‐positive classifications without compromising the prospective detection of incident tears, MSC‐RM mitigates unnecessary preventative burdens in low‐risk individuals while effectively triaging high‐risk patients who would derive genuine benefit from early targeted care.

Evaluating model performance across temporal horizons offers meaningful insights into the longitudinal dynamics of degenerative meniscal tears. The high discriminative accuracy observed for 1‐year rapid‐onset cases (AUC = 0.880) suggests that baseline spatial radiomic features may reflect subtle joint‐level alterations in knees nearing structural failure. From a biomechanical perspective, meniscal degeneration may not follow a strictly linear progression. Rather, our findings are consistent with an accelerating pre‐failure phase: subtle localized matrix disruption and compromised load‐bearing capacity could alter contact stress distribution across the adjacent articular cartilage and subchondral bone. When local physiological compensation is overwhelmed, this progressive mechanical vulnerability may accelerate, ultimately culminating in macroscopic tears detectable on conventional MRI. By detecting these early textural irregularities prior to macroscopic cleft formation, MSC‐RM holds promise for risk stratification, potentially facilitating timely non‐operative interventions within an earlier therapeutic window.

Several considerations warrant discussion regarding the broader implementation of this framework. First, while the 1:1 matched nested case‐control design effectively eliminated confounding by age, sex, and BMI and mitigated severe class imbalance during high‐dimensional radiomic discovery, it inherently adjusted the natural outcome prevalence. Although relative risk stratification and discriminative accuracy (AUC = 0.871, HR = 6.12) are preserved, absolute risk outputs reflect this balanced distribution rather than unselected population rates. Future prospective deployment in unselected clinical screening settings will benefit from baseline risk recalibration via Bayes' rule against real‐world incidence rates. Second, while the OAI cohort provided a high‐quality longitudinal foundation, further validation in diverse, real‐world hospital datasets is essential to ensure generalizability across different scanner vendors and populations. Third, the current reliance on the 3D‐DESS sequence, though optimal for research, suggests a need for future “domain‐agnostic” models that can perform across more ubiquitous 2D clinical sequences. Fourth, although the computational processing is rapid (20–25 s per scan), our model relies on 3D‐DESS MRI. Further validation on conventional 2D clinical sequences is required before broad Picture Archiving and Communication Systems (PACS) integration can be achieved. Finally, while our shallow neural network prioritized transparency, future iterations incorporating explainable AI (XAI) techniques could further illuminate the specific radiomic features—such as bone‐cartilage interface roughness—that contribute most to the risk estimate.

In conclusion, this work establishes a scalable digital framework that extends the role of musculoskeletal imaging from descriptive assessment to prospective risk stratification. By deciphering the subvisual “digital fingerprints” of tissue remodeling, the MSC‐RM framework provides an objective, data‐driven strategy for identifying vulnerable joints. This study supports an evolving paradigm in which quantitative imaging signatures serve as critical tools for standardizing risk communication and informing the next generation of preventive and interventional musculoskeletal research.

## Authorship and Contributorship

All authors were involved in drafting the article or revising it critically for important intellectual content, and all authors approved the final version to be published. Dr **Ting Wang** had full access to all of the data in the study and takes responsibility for the integrity of the data and the accuracy of the data analysis. Study conception and design. **Changhai Ding**, **Ting Wang**. **Juan Wang**, and **Wei Chen**. Acquisition of data. **Shengfa Li**, **Hao Liu**, **Yanbin Zhu**, **Hang Zhang**, and **David Hunter**. Analysis and interpretation of data. **Shengfa Li**, **Hang** **Zhang**, **Qifeng Tao**, **Bin Yu**, **Yang Wang**, **Wenbo Zhao**, **Xin Miao**, **Di Liu**, **Shanshan Yang**, **Tianyu Chen**.

## Author Contributions

Shengfa Li: conceptualization, investigation, funding acquisition, writing – original draft, visualization, validation, methodology, software, formal analysis, resources, data curation. Hao Liu: conceptualization, methodology, software, data curation, formal analysis, investigation, validation, visualization. Yanbin Zhu: conceptualization, methodology, software, data curation, formal analysis, investigation. Hang Zhang: conceptualization, methodology, software, data curation. Qifeng Tao: conceptualization, methodology, software, data curation. Wenbo Zhao: conceptualization, methodology, software, data curation. Xin Miao: conceptualization, methodology, data curation, software. Shanshan Yang: conceptualization, methodology, software, data curation. Tianyu Chen: conceptualization, methodology, software, data curation. David Hunter: writing – review and editing. Changhai Ding: funding acquisition, visualization, supervision, resources, project administration, formal analysis, validation, investigation, writing – review and editing. Ting Wang: investigation, validation, formal analysis, supervision, resources, funding acquisition, visualization, writing – review and editing. Juan Wang: funding acquisition, visualization, project administration, resources, supervision, writing – review and editing. Wei Chen: conceptualization, writing – review and editing, project administration, resources, supervision, validation, visualization, funding acquisition, data curation.

## Funding

This work was supported by the Key development projects of the Sichuan Provincial Science and Technology Plan (Grant No. 2024YFFK0298, S.F.L. was funded; https://kjt.sc.gov.cn/), the Chengdu Medical Research Project (Grant number: 2024487, S.F.L. was funded; 2025246, H.L. was funded; https://cdwjw.chengdu.gov.cn/), the Science and Technology Project of the Chengdu Municipal Science and Technology Bureau (Grant No. 2026‐YF05‐00481‐SN, S.F.L. was funded; https://kjxm.cdkjfw.cn/index.html) and National Natural Science Foundation of China Young Scientists Fund (Class A) (Grant No. 82625041). The funders had no role in study design, data collection and analysis, decision to publish, or preparation of the manuscript.

## Declaration of Interests

The authors declare the following competing interests: David Hunter is the editor of the osteoarthritis section for UpToDate and co‐Editor in Chief of Osteoarthritis and Cartilage. David Hunter provides consulting advice on scientific advisory boards for Haleon, Novartis, Tissuegene, Doron, Sanofi, and Enlivex. All other authors declare no competing interests.

## Ethical Approval

All de‐identified clinical, outcome, and MRI data were obtained from the publicly available Osteoarthritis Initiative (OAI) database, a multicenter prospective cohort designed to identify imaging and biochemical biomarkers of KOA. The OAI protocol was approved by the institutional review boards of all participating centers, including the coordinating site at the University of California, San Francisco (IRB No. 10–00532), and all participants provided written informed consent. As this study involved secondary analysis of anonymized OAI data, no additional ethical approval was required.

## Data Sharing

The data that support the findings of this study are available from the corresponding authors with a signed data access agreement. De­identified patient­level clinical data, outcome data and MRI data are available at https://nda.nih.gov/oai/. The source code for the predictive model is available at https://github.com/Stephen1986Li/POMA23.

## Patient and Public Involvement Statement

Patients or members of the public were not involved in any aspect of this study, including its conception, design, data collection, analysis, interpretation, or dissemination.

## Conflicts of Interest

The authors declare no conflicts of interest.

## Supporting information




**Supporting File 1**: advs78506‐sup‐0001‐SuppMat.docx.

**Supporting File 2**: advs78506‐sup‐0002‐SuppMat.xlsx.

## Data Availability

The data that supports the findings of this study are available in the supplementary material of this article.
