# Supplementary material for: A Multi‐Structural Radiomic Digital Phenotyping Framework for Deciphering Preclinical Evolution of Degenerative Meniscal Tears
Source: Adv Sci (Weinh). 2026 Oct 30:e78506. Online ahead of print. doi: 10.1002/advs.78506 (PMC13721023; doi:10.1002/advs.78506)
Supplement: Supplementary file 1 [file ADVS-9999-e78506-s002.docx]

**Supplementary** Materials

**• Supplementary Figures**

**• Supplementary Tables**

**Figure S1. Segmentation of knee anatomical structures on baseline 3D-DESS MRI.** Representative images illustrating the automatic and manually adjusted segmentation of six anatomical structures used for radiomic feature extraction: femur, femoral cartilage, tibia, tibial cartilage, medial meniscus, and lateral meniscus. Segmentation was initially performed using CNNs based on previously validated frameworks. Manual adjustments were applied using itk-SNAP software by two independent readers blinded to clinical outcomes. VOIs derived from these segmentations served as the basis for radiomic analysis in the DeMTeR cohort. 3D-DESS: 3-Dimensional Double-Echo Steady-State, MRI: Magnetic Resonance Imaging, CNN: Convolutional Neural Networks, VOI: Volumes Of Interest.

**Figure S2. Radiomics feature selection and weighting for single-structure models.** LASSO logistic regression was used to select radiomic features from each anatomical structure for six individual models: (a, g, m) FE-RM, (b, h, n) FC-RM, (c, I, o) TI-RM, (d, j, p) TC-RM, (e, k, q) MM-RM, and (f, I, r) LM-RM. For each model, the selected features with non-zero coefficients are shown, ranked by their absolute contribution weights. LASSO: Least Absolute Shrinkage and Selection Operator, FE-RM: Femur Radiomics Model, FC-RM: Femoral Cartilage Radiomics Model, TI-RM: Tibia Radiomics Model, TC-RM: Tibial Cartilage Radiomics Model, LM-RM: Lateral Meniscus Radiomics Model, MM-RM: Medial Mensicus Radiomics Model.

**Figure S3. Feature selection for multi-structure radiomics models.** LASSO logistic regression identified the most predictive features for (a) the MS-RM, which integrates radiomic features from all six knee structures, and (b) the MSC-RM, which incorporates both radiomics and baseline clinical variables. Each bar represents a non-zero coefficient retained after LASSO selection, with length corresponding to its absolute weight in the model. MSC-RM included 40 radiomic features and 8 clinical features, demonstrating the added value of combining imaging and clinical data for robust prediction of DMTs. LASSO: Least Absolute Shrinkage and Selection Operator, MS-RM: Multi-Structure Radiomics Model, MSC-RM: Multi-Structure and Clinical Radiomics Model, DMTs: Degenerative Meniscus Tears.

**Figure S4. Signal intensity and texture comparison between case and control groups across six knee structures at baseline.** Representative 2D and 3D pixel-level maps from baseline 3D-DESS MRI in case (a) and control knees (b). Visualized structures include the femur, femoral cartilage, tibia, tibial cartilage, medial meniscus, and lateral meniscus. Feature maps were constructed based on average signal intensity (mean voxel values) and spatial texture patterns, using radiomic extraction via the SERA package. Knees in the case group (defined as developing degenerative meniscus tear during the 4-year follow-up based on MOAKS score ≥2) exhibited higher signal intensities and more heterogeneous texture patterns across all six structures compared to controls. These differences highlight early microstructural alterations preceding overt tear detection and support the discriminative capacity of radiomic features for prediction modeling. 2D: 2-Dimensional, 3D: 3-Dimensional, 3D-DESS: 3-Dimensional Double-Echo Steady-State, MRI: Magnetic Resonance Imaging, SERA: Standardized Environment for Radiomics Analysis, MOAKS: Magnetic resonance imaging Osteoarthritis Knee Score.

**Figure S5. Comparative AUC performance of radiomics models versus MOAKS-based models across six knee structures.** ROC curves and corresponding AUC values for radiomics models (red lines) versus their MOAKS-based counterparts (blue lines), based on the test cohort. Subplots display comparisons for: (a) FE-RM vs. FE-MOM, (b) FC-RM vs. FC-MOM, (c) TI-RM vs. TI-MOM, (d) TC-RM vs. TC-MOM, (e) LM-RM vs. LM-MOM, and (f) MM-RM vs. MM-MOM. In several structures (e.g., femur, tibia, tibial cartilage, and medial meniscus), radiomics models significantly outperformed MOAKS-based models in predictive accuracy, as quantified by Delong test p-values. These results support the added prognostic value of radiomics beyond conventional semi-quantitative grading. ROC: Receiver operating characteristic, AUC: Area Under the receiver operating characteristic Curve, MOAKS: Magnetic resonance imaging Osteoarthritis Knee Score, FE-RM: Femur Radiomics Model, FE-MOM: Femur MOAKS Model, FC-RM: Femoral Cartilage, FC-MOM: Femoral Cartilage Radiomics Model, TI-RM: Tibial Radiomics Model, TI-MOM: Tibial MOAKS Model, TC-RM: Tibial Cartilage Radiomics Model. TC-MOM: Tibial Cartilage MOAKS Model, LM-RM: Lateral Meniscus Radiomics Model, LM-MOM: Lateral Meniscus MOAKS Model, MM-RM: Medial Meniscus Radiomics Model, MM-MOM: Medial Meniscus MOAKS Model.

 **Figure S6. Confusion matrices of radiomics and clinical models in the test cohort.** Confusion matrices visualizing classification performance for eight models in predicting incident DMTs within the independent test cohort (n = 138 knees). The models include:(a) FE-RM, (b) FC-RM, (c) TI-RM, (d) TC-RM, (e) LM-RM, (f) MM-RM, (g) MS-RM, and (h) MSC-RM. Each matrix displays the number of true positives, false positives, true negatives, and false negatives. The MSC-RM demonstrated the best overall performance with the highest balanced accuracy and kappa value (κ = 0.560), confirming its superior sensitivity-specificity tradeoff in real-world classification. FE-RM: Femur Radiomics Model, FC-RM: Femoral Cartilage Radiomics Model, TI-RM: Tibia Radiomics Model, TC-RM: Tibial Cartilage Radiomics Model, LM-RM: Lateral Meniscus Radiomics Model, MM-RM: Medial Meniscus Radiomics Model, MS-RM: Multi-Structure Radiomics Model, MSC-RM: Multi-Structure and Clinical Radiomics Model, DMTs: Degenerative Meniscus Tears.

**Figure S7. SHAP model interpretability and compartmental feature attribution of the final MSC-RM model.** (a) SHAP Beeswarm Summary Plot (Top 20 Features). Distribution of impact that the top 20 selected radiomic features exert on the final neural network model’s log-odds output across the test cohort (n = 138). Each point represents an individual knee. Position along the horizontal axis indicates the SHAP value, where positive values denote an increased predicted probability of incident DMTs and negative values denote a decreased probability. The color spectrum reflects the relative feature value (red = high, blue = low). (b) Global Feature Importance Ranking. Ranking of the top 20 predictors sorted by mean absolute SHAP value ("mean "|"SHAP"|) across the test cohort (n = 138). Bars are color-coded according to anatomical joint compartment: MM, LM, TC, FC, FE, TI, and clinical covariates. Corresponding numerical values indicating each feature’s global contribution score are annotated adjacent to the bar ends. (c) Compartmental Attribution Across Knee Load-Bearing Joint Structures. Bar plot illustrating the relative percentage contribution of each anatomical load-bearing structure and clinical covariates to total model predictive attribution. Percentage values representing the aggregate proportion of total model importance are displayed atop each bar. DMTs: Degenerative Meniscal Tears, FC: Femoral Cartilage, FE: Femur, LM: Lateral Meniscus, MM: Medial Meniscus, TC: Tibial Cartilage, TI: Tibia, MSC-RM: Multi-Structure and Clinical Radiomics Model, SHAP: SHapley Additive exPlanations.

**Figure S8. Decision Curve Analysis evaluating the clinical net benefit of the MSC-RM model.** DCA comparing the clinical net benefit of the multivariable spatial compartmental radiomics model (MSC-RM, solid orange line), the clinical model (CM, dashed green line), the “treat-all” strategy (dotted purple line), and the “treat-none” strategy (horizontal solid black line at net benefit = 0) across the independent test cohort (n=138). The horizontal axis depicts the threshold probability ($p_{t}$), and the vertical axis indicates the standardized net benefit. Across a broad range of threshold probabilities ($p_{t}$=0.05-0.85), the MSC-RM model confers consistently superior net benefit compared with default clinical strategies and conventional clinical risk factors alone. CM: clinical model, DCA: Decision Curve Analysis, DMTs: Degenerative Meniscal Tears, MSC-RM: Multi-Structure and Clinical Radiomics Model.

**Figure S9. Longitudinal predictive trajectory and cumulative dynamic AUC analysis across follow-up time horizons in the test cohort.** (a) Cumulative time-horizon AUC comparing the multivariable spatial compartmental radiomics model (MSC-RM, solid orange line with circular markers) and the clinical model (CM, dashed green line with square markers) for predicting incident DMTs within ≤1 year (n = 50), ≤2 years (n = 58), ≤3 years (n = 64), and the full 4-year period (n = 74). Shaded bands represent 95% CI derived from 1,000 bootstrap resamplings. The horizontal dotted gray line denotes chance-level discrimination (AUC = 0.50). (b) Box plots illustrating baseline predicted risk probability distributions across different temporal onset horizons (Year 1 to Year 4) compared to intact control knees (n = 64). Central horizontal lines represent medians, boxes indicate the IQR, and whiskers denote 1.5 $\times$ IQR. AUC: Area Under the receiver operating characteristic Curve, CI: Confidence Interval, CM: Clinical Model, DMTs: Degenerative Meniscal Tears, MSC-RM: Multi-Structure and Clinical Radiomics Model, IQR: interquartile range.

**Table S1. MRI Protocol Details.**

| **MRI parameters** | **3D-DESS** |
| --- | --- |
| Pulse Sequence | de3d1 |
| Plane | Sagittal |
| Fat Sat | WE |
| Matrix (phase) | 307 |
| Matrix (freq) | 384 |
| No. of slices | 160 |
| FOV (mm) | 140 |
| Slice thickness (mm) | 0.7 |
| Skip (mm) | 0 |
| Flip Angle (deg) | 25 |
| TE/TI (ms) | 4.7 |
| TR (ms) | 16.3 |
| BW (Hz/pixel) | 183 |
| Chemical Shift | 0 |
| NAV (NEX) | 1 |
| Echo train length | 1 |
| Phase Encode Axis | A/P |
| Phase Partial Fourier | 1 |
| Readout Partial Fourier | 1 |
| Slice Partial Fourier | 0.75 |
| Distance Factor (%) | 20 |
| Phase Oversampling | 0 |
| Slice Oversampling | 10 |
| Phase Resolution | 80 |
| X-Resolution (mm) | 0.365 |
| Y-Resolution (mm) | 0.456 |
| Scan time (min) | 10.5 |

3D-DESS: sagittal 3-Dimensional Double Echo Steady-State with selective water excitation.

**Table S2. Segmentation accuracy of the automated CNN framework and inter-reader reproducibility on a validation subset.**

| **Segmentation** | **DSCs (n=20)** | | |
| --- | --- | --- | --- |
|  | **CNN vs. Reader 1** | **CNN vs. Reader 2** | **Reader 1 vs. Reader 2** |
| Femur | 0.84 ± 0.04 | 0.85 ± 0.06 | 0.90 ± 0.03 |
| Femoral cartilage | 0.82 ± 0.03 | 0.81 ± 0.02 | 0.88 ± 0.02 |
| Tibia | 0.83 ± 0.03 | 0.87 ± 0.05 | 0.91 ± 0.04 |
| Tibial cartilage | 0.85 ± 0.02 | 0.86 ± 0.04 | 0.89 ± 0.03 |
| Medial meniscus | 0.83 ± 0.02 | 0.84 ± 0.03 | 0.88 ± 0.03 |
| Lateral meniscus | 0.82 ± 0.01 | 0.81 ± 0.05 | 0.87 ± 0.03 |

Data are presented as mean ± SD.

CNN: Convolutional Neural Network, DSCs: Dice Similarity Coefficients, SD: Standard Deviation.

DSCs were good (>0.80).

**Table S3. Baseline characteristics of the participants in the development cohort and test cohort.**

|  | **Development cohort (n = 536)** | **Test cohort (n = 138)** | ***P* value** |
| --- | --- | --- | --- |
| Age (year)^a^ | 61 ± 9 | 59 ± 9 | .055 |
| Female^b^ | 283 (52.8%) | 83 (60.1%) | .123 |
| BMI (kg/m^2^)^a^ | 28.3 ± 4.8 | 27.9 ± 4.4 | .328 |
| Knee injury^b^ | 140 (26.3%) | 40 (29.2%) | .498 |
| Knee surgery^b^ | 58 (10.9%) | 17 (12.3%) | .628 |
| NSAIDs use^b^ | 47 (8.8%) | 5 (3.6%) | .043 |
| Pain medicine use^b^ | 149 (27.9%) | 32 (23.2%) | .266 |
| KOOS pain^c^ | 94 (81, 100) | 89 (78, 100) | .332 |
| KOOS symptom^c^ | 93 (82, 100) | 93 (82, 100) | .959 |
| KOOS funtion, sports, and recreational activities^c^ | 83 (65, 100) | 81 (60, 100) | .601 |
| KOOS quality of life^c^ | 69 (56, 88) | 75 (56, 88) | .408 |

Data are mean ± SD, number (%), or median (IQR).

^a^ Unpaired t-tests are used for differences between means.

^b^ χ2 tests are used for differences between proportions.

^c^ Mann Whitney tests are used for differences between ranks.

BMI: Body Mass Index, NSAIDs: NonSteroidal AntiInflammatory Drugs, KOOS: Knee Outcomes in Osteoarthritis Survey, SD: Standard Deviation, IQR: InterQuartile Range.

**Table S4. Selected features and variables included in the single-structure and integrated predictive models.**

| **Femur radiomic model (n = 7)** ‡ | | |
| --- | --- | --- |
| Class (IBSI/SERA) | Inter-observer ICC (95% CI) | Intra-observer ICC (95% CI) |
| ngl_hdlge_3d_FE | 0.88 (0.81-0.93) | 0.91 (0.85-0.95) |
| ngt_busyness_2d_FE | 0.85 (0.77-0.91) | 0.89 (0.82-0.94) |
| cm_clust_shade_3d_avg_FE | 0.83 (0.74-0.89) | 0.86 (0.78-0.91) |
| ngl_hdlge_2d_FE | 0.87 (0.79-0.92) | 0.90 (0.84-0.94) |
| ngt_complexity_3d_FE | 0.84 (0.76-0.90) | 0.88 (0.81-0.93) |
| ih_min_grad_g_FE | 0.92 (0.86-0.96) | 0.95 (0.90-0.97) |
| ngl_hde_2_5d_FE | 0.86 (0.78-0.92) | 0.89 (0.83-0.94) |
| **Femoral cartilage radiomic model (n = 7)** ‡ | | |
| Class (IBSI/SERA) | Inter-observer ICC (95% CI) | Intra-observer ICC (95% CI) |
| dzm_ldhge_2d_FC | 0.83 (0.75-0.90) | 0.87 (0.80-0.92) |
| morph_vol_dens_mvee_FC | 0.94 (0.89-0.97) | 0.96 (0.92-0.98) |
| cm_joint_max_3d_comb_FC | 0.82 (0.74-0.89) | 0.85 (0.78-0.91) |
| dzm_ldlge_2d_FC | 0.84 (0.76-0.90) | 0.88 (0.81-0.93) |
| ngt_busyness_2d_FC | 0.85 (0.77-0.91) | 0.89 (0.82-0.94) |
| cm_inv_diff_norm_2d_comb_FC | 0.86 (0.78-0.92) | 0.88 (0.81-0.93) |
| ngt_coarseness_2d_FC | 0.81 (0.73-0.88) | 0.84 (0.77-0.90) |
| **Tibia radiomic model (n = 3)** ‡ | | |
| Class (IBSI/SERA) | Inter-observer ICC (95% CI) | Intra-observer ICC (95% CI) |
| cm_joint_max_2d_comb_TI | 0.83 (0.75-0.90) | 0.87 (0.80-0.92) |
| cm_diff_entr_2_5d_avg_TI | 0.85 (0.77-0.91) | 0.88 (0.81-0.93) |
| ivh_auc_TI | 0.91 (0.85-0.95) | 0.94 (0.89-0.97) |
| **Tibial cartilage radiomic model (n = 10)** ‡ | | |
| Class (IBSI/SERA) | Inter-observer ICC (95% CI) | Intra-observer ICC (95% CI) |
| cm_diff_avg_2d_comb_TC | 0.84 (0.76-0.90) | 0.88 (0.81-0.93) |
| cm_inv_var_3d_comb_TC | 0.83 (0.74-0.89) | 0.86 (0.79-0.92) |
| ngl_lde_2_5d_TC | 0.86 (0.78-0.92) | 0.89 (0.82-0.94) |
| morph_pca_elongation_TC | 0.93 (0.87-0.96) | 0.95 (0.91-0.98) |
| ngl_hdlge_2d_TC | 0.87 (0.80-0.92) | 0.90 (0.84-0.94) |
| ngl_dcnu_norm_2_5d_TC | 0.82 (0.74-0.89) | 0.85 (0.77-0.91) |
| cm_sum_entr_2d_comb_TC | 0.85 (0.77-0.91) | 0.88 (0.81-0.93) |
| cm_clust_shade_2_5d_comb_TC | 0.84 (0.76-0.90) | 0.87 (0.80-0.92) |
| cm_sum_entr_3d_avg_TC | 0.86 (0.78-0.92) | 0.89 (0.83-0.94) |
| morph_area_dens_conv_hull_TC | 0.92 (0.86-0.96) | 0.94 (0.89-0.97) |
| **Medial meniscus radiomic model (n = 11)** ‡ | | |
| Class (IBSI/SERA) | Inter-observer ICC (95% CI) | Intra-observer ICC (95% CI) |
| ngl_hde_2_5d_MM | 0.85 (0.77-0.91) | 0.88 (0.81-0.93) |
| ih_min_grad_g_MM | 0.91 (0.85-0.95) | 0.94 (0.89-0.97) |
| ngt_strength_2_5d_MM | 0.83 (0.75-0.90) | 0.87 (0.80-0.92) |
| ih_p10_MM | 0.93 (0.87-0.96) | 0.96 (0.92-0.98) |
| szm_zs_entr_3d_MM | 0.82 (0.74-0.88) | 0.85 (0.78-0.91) |
| ngl_hdlge_3d_MM | 0.87 (0.80-0.93) | 0.91 (0.85-0.95) |
| cm_inv_var_2d_avg_MM | 0.84 (0.76-0.90) | 0.87 (0.80-0.92) |
| morph_area_dens_mvee_MM | 0.92 (0.86-0.96) | 0.95 (0.90-0.97) |
| szm_zs_var_3d_MM | 0.83 (0.75-0.89) | 0.86 (0.79-0.92) |
| morph_area_dens_conv_hull_MM | 0.91 (0.84-0.95) | 0.94 (0.88-0.97) |
| ngt_complexity_2_5d_MM | 0.84 (0.76-0.90) | 0.88 (0.81-0.93) |
| **Lateral meniscus radiomic model (n = 14)** ‡ | | |
| Class (IBSI/SERA) | Inter-observer ICC (95% CI) | Intra-observer ICC (95% CI) |
| morph_vol_dens_mvee_LM | 0.93 (0.87-0.96) | 0.95 (0.91-0.98) |
| morph_vol_dens_aee_LM | 0.92 (0.85-0.95) | 0.94 (0.89-0.97) |
| dzm_zdnu_norm_2d_LM | 0.83 (0.75-0.89) | 0.86 (0.79-0.91) |
| ih_max_grad_g_LM | 0.91 (0.85-0.95) | 0.94 (0.89-0.97) |
| ngl_lde_2_5d_LM | 0.85 (0.77-0.91) | 0.88 (0.81-0.93) |
| dzm_zdnu_2d_LM | 0.84 (0.76-0.90) | 0.87 (0.80-0.92) |
| morph_area_dens_aee_LM | 0.91 (0.84-0.95) | 0.93 (0.88-0.96) |
| ngl_hde_2_5d_LM | 0.86 (0.78-0.92) | 0.89 (0.82-0.94) |
| ngt_strength_2d_LM | 0.82 (0.74-0.89) | 0.86 (0.78-0.91) |
| cm_diff_avg_2d_comb_LM | 0.84 (0.76-0.90) | 0.87 (0.80-0.92) |
| dzm_sdhge_2_5d_LM | 0.83 (0.75-0.90) | 0.86 (0.79-0.92) |
| morph_area_dens_mvee_LM | 0.93 (0.87-0.96) | 0.95 (0.91-0.98) |
| szm_gl_var_2d_LM | 0.82 (0.74-0.88) | 0.85 (0.78-0.91) |
| cm_info_corr2_2d_avg_LM | 0.85 (0.77-0.91) | 0.88 (0.82-0.93) |
| **Multi-structure radiomic model (n = 41)** ‡ | | |
| cm_diff_avg_2d_comb_TC | | |
| dzm_zdnu_2d_LM | | |
| morph_vol_dens_mvee_FC | | |
| ngl_hdlge_3d_FE | | |
| ngl_hdlge_3d_MM | | |
| ngt_busyness_2d_FE | | |
| morph_area_dens_conv_hull_MM | | |
| cm_clust_shade_2_5d_comb_TC | | |
| ngt_strength_2_5d_MM | | |
| morph_pca_elongatcon_TC | | |
| cm_joint_max_2d_comb_TI | | |
| ngt_busyness_2d_FC | | |
| cm_inv_var_2d_avg_MM | | |
| ngl_hdlge_2d_FE | | |
| ih_min_grad_g_MM | | |
| dzm_zdnu_norm_2d_LM | | |
| ih_min_grad_g_FE | | |
| morph_area_dens_mvee_LM | | |
| morph_area_dens_conv_hull_TC | | |
| morph_vol_dens_aee_LM | | |
| morph_vol_dens_mvee_LM | | |
| ngl_hdlge_2d_TC | | |
| cm_inv_var_3d_comb_TC | | |
| cm_diff_entr_2_5d_avg_TI | | |
| cm_sum_entr_3d_avg_TC | | |
| szm_gl_var_2d_LM | | |
| ih_p10_MM | | |
| cm_clust_shade_3d_avg_FE | | |
| szm_zs_entr_3d_MM | | |
| ngl_lde_2_5d_LM | | |
| ih_max_grad_g_LM | | |
| ngl_hde_2_5d_LM | | |
| ngt_complexity_3d_FE | | |
| dzm_ldlge_2d_FC | | |
| morph_area_dens_aee_LM | | |
| cm_diff_avg_2d_comb_LM | | |
| cm_inv_diff_norm_2d_comb_FC | | |
| cm_joint_max_3d_comb_FC | | |
| szm_zs_var_3d_MM | | |
| ngl_hde_2_5d_MM | | |
| cm_sum_entr_2d_comb_TC | | |
| **Multi-structure and clinical variable radiomic model (n = 48)** ‡ | | |
| cm_diff_avg_2d_comb_TC | | |
| ngl_hde_2_5d_LM | | |
| morph_vol_dens_mvee_FC | | |
| morph_pca_elongatcon_TC | | |
| cm_clust_shade_2_5d_comb_TC | | |
| ngt_strength_2_5d_MM | | |
| morph_area_dens_conv_hull_MM | | |
| Race | | |
| cm_sum_entr_3d_avg_TC | | |
| dzm_zdnu_2d_LM | | |
| ngl_hdlge_3d_MM | | |
| cm_diff_entr_2_5d_avg_TI | | |
| ih_min_grad_g_FE | | |
| ngt_busyness_2d_FE | | |
| morph_area_dens_aee_LM | | |
| morph_vol_dens_mvee_LM | | |
| ngl_hdlge_2d_FE | | |
| ngl_hdlge_3d_FE | | |
| ngl_hde_2_5d_FE | | |
| morph_vol_dens_aee_LM | | |
| dzm_ldlge_2d_FC | | |
| ih_min_grad_g_MM | | |
| morph_area_dens_mvee_MM | | |
| cm_joint_max_2d_comb_TI | | |
| ngt_busyness_2d_FC | | |
| ngl_lde_2_5d_LM | | |
| ngt_coarseness_2d_FC | | |
| morph_area_dens_conv_hull_TC | | |
| cm_inv_var_3d_comb_TC | | |
| szm_gl_var_2d_LM | | |
| dzm_zdnu_norm_2d_LM | | |
| Baseline body mass index | | |
| cm_clust_shade_3d_avg_FE | | |
| ngl_hdlge_2d_TC | | |
| szm_zs_var_3d_MM | | |
| Gender | | |
| ih_p10_MM | | |
| cm_joint_max_3d_comb_FC | | |
| Baseline glucosamine use | | |
| Baseline KOOS knee pain score | | |
| szm_zs_entr_3d_MM | | |
| Baseline physical activity scale for the elderly score | | |
| morph_area_dens_mvee_LM | | |
| cm_inv_var_2d_avg_MM | | |
| dzm_sdhge_2_5d_LM | | |
| Baseline KOOS quality of life score | | |
| Baseline KOOS symptoms score | | |
| ngt_complexity_2_5d_MM | | |
| **Clinical model (n = 8)** | | |
| Race | | |
| Baseline body mass index | | |
| Gender | | |
| Baseline glucosamine use | | |
| Baseline physical activity scale for the elderly score | | |
| Baseline KOOS knee pain score | | |
| Baseline KOOS symptoms score | | |
| Baseline KOOS quality of life score | | |
| **Femur MOAKS Model (n = 12)** | | |
| Baseline BML (% lesion that is edema) - femur medial anterior (trochlear) | | |
| Baseline BML (% lesion that is edema) - femur lateral anterior (trochlear) | | |
| Baseline BML (% lesion that is edema) - femur medial central | | |
| Baseline BML (% lesion that is edema) - femur lateral central | | |
| Baseline BML (% lesion that is edema) - femur medial posterior | | |
| Baseline BML (% lesion that is edema) - femur lateral posterior | | |
| Baseline osteophyte size - femur medial anterior (trochlear) | | |
| Baseline osteophyte size - femur lateral anterior (trochlear) | | |
| Baseline osteophyte size - femur medial posterior | | |
| Baseline osteophyte size - femur lateral posterior | | |
| Baseline osteophyte size - femur medial central | | |
| Baseline osteophyte size - femur lateral central | | |
| **Femoral cartilage MOAKS Model (n = 6)** | | |
| Baseline cartilage morphology - femur medial anterior (trochlear) | | |
| Baseline cartilage morphology - femur lateral anterior (trochlear) | | |
| Baseline cartilage morphology - femur medial posterior | | |
| Baseline cartilage morphology - femur lateral posterior | | |
| Baseline cartilage morphology - femur medial central | | |
| Baseline cartilage morphology - femur lateral central | | |
| **Tibia MOAKS Model (n =9)** | | |
| Baseline BML (% lesion that is edema) - tibia sub-spinous | | |
| Baseline BML (% lesion that is edema) - tibia medial anterior | | |
| Baseline BML (% lesion that is edema) - tibia lateral anterior | | |
| Baseline BML (% lesion that is edema) - tibia medial central | | |
| Baseline BML (% lesion that is edema) - tibia lateral central | | |
| Baseline BML (% lesion that is edema) - tibia medial posterior | | |
| Baseline BML (% lesion that is edema) - tibia lateral posterior | | |
| Baseline osteophyte size - tibia medial | | |
| Baseline osteophyte size - tibial lateral | | |
| **Tibial cartilage MOAKS Model (n = 6)** | | |
| Baseline cartilage morphology - tibia medial anterior | | |
| Baseline cartilage morphology - tibia lateral anterior | | |
| Baseline cartilage morphology - tibia medial central | | |
| Baseline cartilage morphology - tibia lateral central | | |
| Baseline cartilage morphology - tibia medial posterior | | |
| Baseline cartilage morphology - tibia lateral posterior | | |
| **Medial meniscus MOAKS Model (n=12)** | | |
| Baseline medial meniscal morphology - anterior horn | | |
| Baseline medial meniscal morphology - body | | |
| Baseline medial meniscal morphology - posterior horn | | |
| Baseline medial meniscal hypertrophy - anterior horn | | |
| Baseline medial meniscal hypertrophy - body | | |
| Baseline medial meniscal hypertrophy - posterior horn | | |
| Baseline medial meniscal signal abnormality - anterior horn | | |
| Baseline medial meniscal signal abnormality - body | | |
| Baseline medial meniscal signal abnormality - posterior horn | | |
| Baseline medial meniscal extrusion - medially | | |
| Baseline medial meniscal extrusion - anteriorly | | |
| Baseline medial meniscal morphology - posterior root tear | | |
| **Lateral meniscus MOAKS Model (n = 12)** | | |
| Baseline lateral meniscal morphology - anterior horn | | |
| Baseline lateral meniscal morphology - body | | |
| Baseline lateral meniscal morphology - posterior horn | | |
| Baseline lateral meniscal hypertrophy - anterior horn | | |
| Baseline lateral meniscal hypertrophy - body | | |
| Baseline lateral meniscal hypertrophy - posterior horn | | |
| Baseline lateral meniscal signal abnormality - anterior horn | | |
| Baseline lateral meniscal signal abnormality - body | | |
| Baseline lateral meniscal signal abnormality - posterior horn | | |
| Baseline lateral meniscal extrusion - anteriorly | | |
| Baseline lateral meniscal extrusion - laterally | | |
| Baseline lateral meniscal morphology - posterior root tear | | |
| **Multi-structure MOAKS Model (n= 57)** | | |
| Baseline BML (% lesion that is edema) - femur medial anterior (trochlear) | | |
| Baseline BML (% lesion that is edema) - femur lateral anterior (trochlear) | | |
| Baseline BML (% lesion that is edema) - femur medial central | | |
| Baseline BML (% lesion that is edema) - femur lateral central | | |
| Baseline BML (% lesion that is edema) - femur medial posterior | | |
| Baseline BML (% lesion that is edema) - femur lateral posterior | | |
| Baseline osteophyte size - femur medial anterior (trochlear) | | |
| Baseline osteophyte size - femur lateral anterior (trochlear) | | |
| Baseline osteophyte size - femur medial posterior | | |
| Baseline osteophyte size - femur lateral posterior | | |
| Baseline osteophyte size - femur medial central | | |
| Baseline osteophyte size - femur lateral central | | |
| Baseline cartilage morphology - femur medial anterior (trochlear) | | |
| Baseline cartilage morphology - femur lateral anterior (trochlear) | | |
| Baseline cartilage morphology - femur medial posterior | | |
| Baseline cartilage morphology - femur lateral posterior | | |
| Baseline cartilage morphology - femur medial central | | |
| Baseline cartilage morphology - femur lateral central | | |
| Baseline BML (% lesion that is edema) - tibia sub-spinous | | |
| Baseline BML (% lesion that is edema) - tibia medial anterior | | |
| Baseline BML (% lesion that is edema) - tibia lateral anterior | | |
| Baseline BML (% lesion that is edema) - tibia medial central | | |
| Baseline BML (% lesion that is edema) - tibia lateral central | | |
| Baseline BML (% lesion that is edema) - tibia medial posterior | | |
| Baseline BML (% lesion that is edema) - tibia lateral posterior | | |
| Baseline osteophyte size - tibia medial | | |
| Baseline osteophyte size - tibial lateral | | |
| Baseline cartilage morphology - tibia medial anterior | | |
| Baseline cartilage morphology - tibia lateral anterior | | |
| Baseline cartilage morphology - tibia medial central | | |
| Baseline cartilage morphology - tibia lateral central | | |
| Baseline cartilage morphology - tibia medial posterior | | |
| Baseline cartilage morphology - tibia lateral posterior | | |
| Baseline lateral meniscal morphology - anterior horn | | |
| Baseline lateral meniscal morphology - body | | |
| Baseline lateral meniscal morphology - posterior horn | | |
| Baseline lateral meniscal hypertrophy - anterior horn | | |
| Baseline lateral meniscal hypertrophy - body | | |
| Baseline lateral meniscal hypertrophy - posterior horn | | |
| Baseline lateral meniscal signal abnormality - anterior horn | | |
| Baseline lateral meniscal signal abnormality - body | | |
| Baseline lateral meniscal signal abnormality - posterior horn | | |
| Baseline lateral meniscal extrusion - anteriorly | | |
| Baseline lateral meniscal extrusion - laterally | | |
| Baseline lateral meniscal morphology - posterior root tear | | |
| Baseline medial meniscal morphology - anterior horn | | |
| Baseline medial meniscal morphology - body | | |
| Baseline medial meniscal morphology - posterior horn | | |
| Baseline medial meniscal hypertrophy - anterior horn | | |
| Baseline medial meniscal hypertrophy - body | | |
| Baseline medial meniscal hypertrophy - posterior horn | | |
| Baseline medial meniscal signal abnormality - anterior horn | | |
| Baseline medial meniscal signal abnormality - body | | |
| Baseline medial meniscal signal abnormality - posterior horn | | |
| Baseline medial meniscal extrusion - medially | | |
| Baseline medial meniscal extrusion - anteriorly | | |
| **Multi-structure and clinical variable MOAKS Model (n = 65)** | | |
| Baseline BML (% lesion that is edema) - femur medial anterior (trochlear) | | |
| Baseline BML (% lesion that is edema) - femur lateral anterior (trochlear) | | |
| Baseline BML (% lesion that is edema) - femur medial central | | |
| Baseline BML (% lesion that is edema) - femur lateral central | | |
| Baseline BML (% lesion that is edema) - femur medial posterior | | |
| Baseline BML (% lesion that is edema) - femur lateral posterior | | |
| Baseline osteophyte size - femur medial anterior (trochlear) | | |
| Baseline osteophyte size - femur lateral anterior (trochlear) | | |
| Baseline osteophyte size - femur medial posterior | | |
| Baseline osteophyte size - femur lateral posterior | | |
| Baseline osteophyte size - femur medial central | | |
| Baseline osteophyte size - femur lateral central | | |
| Baseline cartilage morphology - femur medial anterior (trochlear) | | |
| Baseline cartilage morphology - femur lateral anterior (trochlear) | | |
| Baseline cartilage morphology - femur medial posterior | | |
| Baseline cartilage morphology - femur lateral posterior | | |
| Baseline cartilage morphology - femur medial central | | |
| Baseline cartilage morphology - femur lateral central | | |
| Baseline BML (% lesion that is edema) - tibia sub-spinous | | |
| Baseline BML (% lesion that is edema) - tibia medial anterior | | |
| Baseline BML (% lesion that is edema) - tibia lateral anterior | | |
| Baseline BML (% lesion that is edema) - tibia medial central | | |
| Baseline BML (% lesion that is edema) - tibia lateral central | | |
| Baseline BML (% lesion that is edema) - tibia medial posterior | | |
| Baseline BML (% lesion that is edema) - tibia lateral posterior | | |
| Baseline osteophyte size - tibia medial | | |
| Baseline osteophyte size - tibial lateral | | |
| Baseline cartilage morphology - tibia medial anterior | | |
| Baseline cartilage morphology - tibia lateral anterior | | |
| Baseline cartilage morphology - tibia medial central | | |
| Baseline cartilage morphology - tibia lateral central | | |
| Baseline cartilage morphology - tibia medial posterior | | |
| Baseline cartilage morphology - tibia lateral posterior | | |
| Baseline lateral meniscal morphology - anterior horn | | |
| Baseline lateral meniscal morphology - body | | |
| Baseline lateral meniscal morphology - posterior horn | | |
| Baseline lateral meniscal hypertrophy - anterior horn | | |
| Baseline lateral meniscal hypertrophy - body | | |
| Baseline lateral meniscal hypertrophy - posterior horn | | |
| Baseline lateral meniscal signal abnormality - anterior horn | | |
| Baseline lateral meniscal signal abnormality - body | | |
| Baseline lateral meniscal signal abnormality - posterior horn | | |
| Baseline lateral meniscal extrusion - anteriorly | | |
| Baseline lateral meniscal extrusion - laterally | | |
| Baseline lateral meniscal morphology - posterior root tear | | |
| Baseline medial meniscal morphology - anterior horn | | |
| Baseline medial meniscal morphology - body | | |
| Baseline medial meniscal morphology - posterior horn | | |
| Baseline medial meniscal hypertrophy - anterior horn | | |
| Baseline medial meniscal hypertrophy - body | | |
| Baseline medial meniscal hypertrophy - posterior horn | | |
| Baseline medial meniscal signal abnormality - anterior horn | | |
| Baseline medial meniscal signal abnormality - body | | |
| Baseline medial meniscal signal abnormality - posterior horn | | |
| Baseline medial meniscal extrusion - medially | | |
| Baseline medial meniscal extrusion - anteriorly | | |
| Baseline medial meniscal morphology - posterior root tear | | |
| Race | | |
| Baseline BMI | | |
| Gender | | |
| Baseline glucosamine use | | |
| Baseline physical activity scale for the elderly score | | |
| Baseline KOOS knee pain score | | |
| Baseline KOOS symptoms score | | |
| Baseline KOOS quality of life score | | |

‡ Selected radiomics feature name = Radiomics feature name + anatomical structure.

FE: Femur, FC: Femoral Cartilage, TI: Tibia, TC: Tibial Cartilage, LM: Lateral Meniscus, MM: Medial Meniscus, KOOS: Knee Outcomes in Osteoarthritis Survey, MOAKS: Magnetic resonance imaging Osteoarthritis Knee Score, BML: Bone Marrow Lesion, ICC: Intraclass Correlation Coefficient, CI: Confidence Interval.

Radiomic feature naming follows the Standardized Environment for Radiomics Analysis (SERA) and Image Biomarker Standardization Initiative (IBSI) conventions: feature class + metric name + extraction dimension (2D, 2.5D, 3D) + anatomical structure. ICC estimates were calculated using a two-way random-effects model evaluating absolute agreement on a randomly chosen validation cohort knee MRI (n = 20). Intra-observer agreement was assessed by one observer re-evaluating cases after a 4-week interval. All features satisfied the pre-specified robustness criterion of both intra- and inter-observer ICC ≥ 0.80.

**Table S5. Prediction performance of single-structure radiomic models in the test cohort.**

| **Model name** | **Development cohort** | **Test cohort** |
| --- | --- | --- |
| **FE-RM** |  |  |
| Sensitivity | 65.4% (172/263) | 71.6% (53/74) |
| Specificity | 52.7% (144/273) | 54.7% (35/64) |
| AUC | 0.613 (0.565-0.659) | 0.717 (0.625-0.794) |
| **FE-MOM** |  |  |
| Sensitivity | 24.3% (64/263) | 17.6% (13/74) |
| Specificity | 96.0% (262/273) | 93.8% (60/64) |
| AUC | 0.606 (0.578-0.634) | 0.561 (0.508-0.613) |
| **FC-RM** |  |  |
| Sensitivity | 65.8% (173/263) | 73.0% (54/74) |
| Specificity | 38.1% (104/273) | 35.9% (23/64) |
| AUC | 0.563 (0.514-0.611) | 0.575 (0.477-0.666) |
| **FC-MOM** |  |  |
| Sensitivity | 51.7% (136/263) | 43.2% (32/74) |
| Specificity | 90.5% (247/273) | 82.8% (53/64) |
| AUC | 0.724 (0.688-0.758) | 0.635 (0.558-0.706) |
| **TI-RM** |  |  |
| Sensitivity | 67.3% (177/263) | 78.4% (58/74) |
| Specificity | 46.2% (126/273) | 46.9% (30/64) |
| AUC | 0.613 (0.565-0.659) | 0.672 (0.575-0.756) |
| **TI-MOM** |  |  |
| Sensitivity | 24.3% (64/263) | 17.6% (13/74) |
| Specificity | 95.6% (261/273) | 93.8% (60/64) |
| AUC | 0.603 (0.574-0.631) | 0.560 (0.506-0.611) |
| **TC-RM** |  |  |
| Sensitivity | 68.4% (180/263) | 67.6% (50/74) |
| Specificity | 68.1% (186/273) | 64.1% (41/64) |
| AUC | 0.748 (0.706-0.787) | 0.713 (0.621-0.791) |
| **TC-MOM** |  |  |
| Sensitivity | 48.7% (128/263) | 43.2% (32/74) |
| Specificity | 89.7% (245/273) | 82.8% (53/64) |
| AUC | 0.704 (0.667-0.739) | 0.654 (0.579-0.722) |
| **MM-RM** |  |  |
| Sensitivity | 68.8% (181/263) | 75.7% (56/74) |
| Specificity | 58.6% (160/273) | 64.1% (41/64) |
| AUC | 0.669 (0.662-0.713) | 0.750 (0.567-0.825) |
| **MM-MOM** |  |  |
| Sensitivity | 51.7% (136/263) | 43.2% (32/74) |
| Specificity | 89.7% (245/273) | 81.3% (52/64) |
| AUC | 0.723 (0.687-0.757) | 0.635 (0.557-0.705) |
| **LM-RM** |  |  |
| Sensitivity | 48.7% (128/263) | 52.7% (39/74) |
| Specificity | 59.7% (163/273) | 65.6% (42/64) |
| AUC (95% CI) | 0.564 (0.515-0.611) | 0.582 (0.484-0.675) |
| **LM-MOM** |  |  |
| Sensitivity | 51.7% (136/263) | 43.2% (32/74) |
| Specificity | 89.7% (245/273) | 81.3% (52/64) |
| AUC | 0.715 (0.679-0.749) | 0.637 (0.561-0.708) |

Data are percentages (numerator/denominator for percentages) or mean (95% CI).

AUC: Area Under the receiver operating characteristic Curve, CI: Confidence Interval, FE-RM: Femur Radiomic Model, FC-RM: Femoral Cartilage Radiomic Model, TI-RM: Tibia Radiomic Model, TC-RM: Tibial Cartilage Radiomic Model, LM-RM: Lateral Meniscus Radiomic Model, MM-RM: Medial Meniscus Radiomic Model, FE-MOM: Femur MOAKS Model, FC-MOM: Femoral Cartilage MOAKS Model, TI-MOM: Tibia MOAKS Model, TC-MOM: Tibial Cartilage MOAKS Model, LM-MOM: Lateral Meniscus MOAKS Model, MM-MOM: Medial Meniscus MOAKS Model, MOAKS: Magnetic resonance imaging Osteoarthritis Knee Score, sensitivity = TP / (TP + FN), specificity = TN / (TN + FP), TP: True Positive, TN: True Negitive, FP: False Positive, FN: False Negitive.

**Table S6. Comparing the areas under two correlated ROC curves between models.**

| **Model item** | **Development cohort** | |  | **Test cohort** | |
| --- | --- | --- | --- | --- | --- |
|  | **AUC difference** | ***P* value** |  | **AUC difference** | ***P* value** |
| **SS-RM vs. MOAKS** |  |  |  |  |  |
| FE-RM vs. FE-MOM | 0.007 [(-0.045)-0.059] | .800 |  | 0.156 (0.063-0.249) | <.001 |
| FC-RM vs. FC-MOM | -0.161 [(-0.223)-(-0.100)] | <.001 |  | -0.061 [(-0.188)-0.067] | .353 |
| TI-RM vs. TI-MOM | 0.010 [(-0.046)-0.066] | .720 |  | 0.112 (0.006-0.218) | .039 |
| TC-RM vs. TC-MOM | 0.044 [(-0.010-0.099] | .110 |  | 0.060 [(-0.063)-0.182] | .340 |
| MM-RM vs. MM-MOM | -0.054 [(-0.114)-0.006] | .078 |  | 0.116 (0.0004-0.231) | .049 |
| LM-RM vs. LM-MOM | -0.151 [(-0.210-(-0.093)) | <.001 |  | -0.055 [(-0.176)-0.066] | .373 |
| **MS-RM vs. CM/ MOAKS** |  |  |  |  |  |
| MS-RM vs. CM | 0.330 (0.274-0.385) | <.001 |  | 0.324 (0.199-0.450) | <.001 |
| MS-RM vs. FE-MOM | 0.288 (0.248-0.328) | <.001 |  | 0.280 (0.191-0.370) | <.001 |
| MS-RM vs. FC-MOM | 0.170 (0.126-0.214) | <.001 |  | 0.206 (0.103-0.309) | <.001 |
| MS-RM vs. TI-MOM | 0.292 (0.252-0.332) | <.001 |  | 0.281 (0.192-0.371) | <.001 |
| MS-RM vs. TC-MOM | 0.190 (0.146-0.235) | <.001 |  | 0.187 (0.086-0.288) | <.001 |
| MS-RM vs. MM-MOM | 0.171 (0.127-0.216) | <.001 |  | 0.206 (0.104-0.309) | <.001 |
| MS-RM vs. LM-MOM | 0.180 (0.135-0.224) | <.001 |  | 0.204 (0.103-0.305) | <.001 |
| MS-RM vs. MS-MOM | 0.159 (0.116-0.203) | <.001 |  | 0.210 (0.113-0.307) | <.001 |
| MS-RM vs. MSC-MOM | 0.118 (0.072-0.164) | <.001 |  | 0.155 (0.046-0.264) | .005 |
| **MS-RM vs. SS-RM** |  |  |  |  |  |
| MS-RM vs. FE-RM | 0.282 (0.232-0.331) | <.001 |  | 0.124 (0.034-0.215) | .007 |
| MS-RM vs. FC-RM | 0.332 (0.281-0.383) | <.001 |  | 0.266 (0.156-0.377) | <.001 |
| MS-RM vs. TI-RM | 0.281 (0.230-0.333) | <.001 |  | 0.169 (0.066-0.273) | <.001 |
| MS-RM vs. TC-RM | 0.146 (0.111-0.181) | <.001 |  | 0.128 (0.037-0.218) | .006 |
| MS-RM vs. MM-RM | 0.226 (0.183-0.268) | <.001 |  | 0.091 (0.014-0.168) | .021 |
| MS-RM vs. LM-RM | 0.331 (0.280-0.382) | <.001 |  | 0.259 (0.155-0.362) | <.001 |
| **MSC-RM vs. CM/ MOAKS** |  |  |  |  |  |
| MSC-RM vs. CM | 0.325 (0.273-0.376) | <.001 |  | 0.354 (0.247-0.461) | <.001 |
| MSC-RM vs. FE-MOM | 0.283 (0.244-0.322) | <.001 |  | 0.310 (0.230-0.389) | <.001 |
| MSC-RM vs. FC-MOM | 0.165 (0.122-0.209) | <.001 |  | 0.236 (0.141-0.330) | <.001 |
| MSC-RM vs. TI-MOM | 0.287 (0.248-0.326) | <.001 |  | 0.311 (0.232-0.390) | <.001 |
| MSC-RM vs. TC-MOM | 0.185 (0.141-0.230) | <.001 |  | 0.217 (0.125-0.309) | <.001 |
| MSC-RM vs. MM-MOM | 0.167 (0.123-0.210) | <.001 |  | 0.236 (0.142-0.330) | <.001 |
| MSC-RM vs. LM-MOM | 0.175 (0.130-0.219) | <.001 |  | 0.234 (0.141-0.326) | <.001 |
| MSC-RM vs. MS-MOM | 0.325 (0.273-0.376) | <.001 |  | 0.354 (0.247-0.461) | <.001 |
| MSC-RM vs. MSC-MOM | 0.283 (0.244-0.322) | <.001 |  | 0.310 (0.230-0.389) | <.001 |
| **MSC-RM vs. SS-RM** |  |  |  |  |  |
| MSC-RM vs. FE-RM | 0.277 (0.226-0.327) | <.001 |  | 0.154 (0.060-0.248) | .0013 |
| MSC-RM vs. FC-RM | 0.327 (0.273-0.380) | <.001 |  | 0.296 (0.193-0.399) | <.001 |
| MSC-RM vs. TI-RM | 0.276 (0.225-0.328) | <.001 |  | 0.199 (0.100-0.298) | <.001 |
| MSC-RM vs. TC-RM | 0.141 (0.104-0.178) | .002 |  | 0.157 (0.072-0.242) | <.001 |
| MSC-RM vs. MM-RM | 0.221 (0.177-0.264) | <.001 |  | 0.121 (0.035-0.207) | <.001 |
| MSC-RM vs. LM-RM | 0.326 (0.275-0.378) | .004 |  | 0.288 (0.181-0.396) | <.001 |
| **MS-RM vs. MSC-RM** | 0.005 [(-0.018)-0.028] | .678 |  | -0.030 [(-0.091)-0.032] | .344 |

Data are mean (95% CI).

ROC: Receiver Operating Characteristic, AUC: Area under the ROC Curve, CI: Confidence Interval, FE-RM: Femur Radiomic Model, FC-RM: Femoral Cartilage Radiomic Model, TI-RM: Tibia Radiomic Model, TC-RM: Tibial Cartilage Radiomic Model, LM-RM: Lateral Meniscus Radiomic Model, MM-RM: Medial Meniscus Radiomic Model, SS-RM: Single-Structure Radiomic Model, MS-RM: Multi-Structure Radiomic Model, MSC-RM: Multi-Structure and Clinical variable Radiomic Model, FE-MOM: Femur MOAKS Model, FC-MOM: Femoral Cartilage MOAKS Model, TI-MOM: Tibia MOAKS Model, TC-MOM: Tibial Cartilage MOAKS Model, LM-MOM: Lateral Meniscus MOAKS Model, MM-MOM: Medial Meniscus MOAKS Model, MS-MOM: Multi-Structure MOAKS Model, MSC-MOM: Multi-Structure and Clinical variable MOAKS Model, MOAKS: Magnetic resonance imaging Osteoarthritis knee Score, CM: Clinical Model.

**Table S7. Related risks of outcomes for predictive model outputs.**

| **Predicting models** | **Crude OR** | ***P* value^†^** | **Adjusted OR^‡^** | ***P* value^†^** |
| --- | --- | --- | --- | --- |
| FE-RM | 2.276 (1.670-3.102) | <.001 | 2.424 (1.741-3.374) | <.001 |
| FC-RM | 1.248 (0.908-1.716) | .173 | 1.250 (0.901-1.734) | 0.182 |
| TI-RM | 1.986 (1.450-2.719) | <.001 | 1.971 (1.426-2.724) | <.001 |
| TC-RM | 4.436 (3.216-6.119) | <.001 | 4.416 (3.184-6.122) | <.001 |
| MM-RM | 3.503 (2.543-4.825) | <.001 | 3.531 (2.514-4.959) | <.001 |
| LM-RM | 1.526 (1.124-2.070) | .007 | 1.608 (1.173-2.204) | .003 |
| MS-RM | 17.850 (12.160-26.204) | <.001 | 19.241 (12.859-28.790) | <.001 |
| CM | 1.707 (1.112-2.621) | .014 | 1.879 (1.009-3.500) | .047 |
| MSC-RM | 16.870 (11.448-24.859) | <.001 | 19.740 (12.883-30.248) | <.001 |

Data are OR (95% CI).

^†^GEE was used to assess the significance levels of individual risk factors, the outputs of predictive models were independent variables, meniscus injury incidence was dependent variable.

^‡^ Adjustment for baseline age, sex, BMI, knee side, race, and KOOS knee pain score.

OR: Odds Ratio, BMI: Body Mass Index, GEE: Generalized Estimating Equation, CI: Confidence Interval, FE-RM: Femur Radiomic Model, FC-RM: Femoral Cartilage Radiomic Model, TI-RM: Tibia Radiomic Model, TC-RM: Tibial Cartilage Radiomic Model, LM-RM: Lateral Meniscus Radiomic Model, MM-RM: Medial Meniscus Radiomic Model, MS-RM: Multi-Structure Radiomic Model, MSC-RM: Multi-Structure and Clinical variable Radiomics Model, KOOS: Knee Outcomes in Osteoarthritis Survey, MOAKS: Magnetic resonance imaging Osteoarthritis knee Score, CM: Clinical Model.

**Table S8. Predictive performance of resident surgeons under the assistance of MSC-RM.**

|  | **Test cohort** | |
| --- | --- | --- |
|  | **No** | **Yes** |
| **Resident surgeon 1** |  |  |
| Sensitivity | 47.3% (35/74) | 77.0% (57/74) |
| Specificity | 31.3% (20/64) | 76.6% (49/64) |
| **Resident surgeon 2** |  |  |
| Sensitivity | 40.5% 30/74) | 81.1% (60/74) |
| Specificity | 57.8% (37/64) | 70.3% (45/64) |
| **Resident surgeon 3** |  |  |
| Sensitivity | 23.0% (17/74) | 74.3% (55/74) |
| Specificity | 39.1% (25/64) | 85.9% (55/64) |
| **Resident surgeon 4** |  |  |
| Sensitivity | 13.5% (10/74) | 71.6% (53/74) |
| Specificity | 26.6% (17/64) | 81.3% (52/64) |
| **Resident surgeon 5** |  |  |
| Sensitivity | 27.0% (20/74) | 67.6% (50/74) |
| Specificity | 46.9% (30/64) | 65.6% (42/64) |

Data are percentages (numerator/denominator for percentages). Clinical practice years of resident surgeons: 1 to 4 years.

MSC-RM: Multi-Structure and Clinical variable Radiomic Model, sensitivity = TP / (TP + FN), specificity = TN / (TN + FP), TP: True positive, TN: True negative, FP: False positive, FN: False negative.

**Table S9. Predictive performance of resident surgeons under the assistance of MSC-RM.**

|  | **Resident surgeons (n = 5)** | | |
| --- | --- | --- | --- |
| **Test cohort** | **Without MSC-RM support** | **With MSC-RM support** | ***P* value** |
| Sensitivity | 30.3% (13.4%-47.2%) | 74.3% (67.9%-80.7%) | <.001 |
| Specificity | 40.3% (24.8%-55.8%) | 75.9% (65.8%-86.1%) | <.001 |

Data are mean (95% CI). Clinical practice years of resident surgeons: 1 to 4 years.

CI: Confidence interval, MSC-RM: Multi-Structure and Clinical variable Radiomic Model.

**Table S10. Cohort-level comparison of representative 3D-DESS radiomic features across load-bearing knee compartments between incident DMT cases and matched controls**

| **Radiomic Feature** | **Compartment** | **Controls (n=337)** | **Cases (n=337)** | ***t* statistic** | ***P* value** |
| --- | --- | --- | --- | --- | --- |
| **Intensity Histogram Mean** |  |  |  |  |  |
| (ih_mean) |  |  |  |  |  |
|  | FE | 66.7 ± 2.5 | 66.2 ± 2.2 | -2.57 | 0.010 |
|  | FC | 63.3 ± 1.4 | 63.2 ± 1.8 | -0.60 | 0.548 |
|  | TI | 63.5 ± 4.0 | 63.9 ± 3.8 | 1.41 | 0.158 |
|  | TC | 54.1 ± 4.7 | 54.4 ± 4.0 | 1.12 | 0.265 |
|  | MM | 52.4 ± 4.5 | 53.8 ± 3.8 | 4.32 | < 0.001 |
|  | LM | 52.1 ± 2.8 | 52.2 ± 2.8 | 0.66 | 0.509 |
| **3D Joint Entropy** |  |  |  |  |  |
| (cm_joint_entr_3d_avg) |  |  |  |  |  |
|  | FE | 11.2 ± 0.3 | 11.2 ± 0.3 | -0.53 | 0.599 |
|  | FC | 11.5 ± 0.4 | 11.5 ± 0.4 | 0.34 | 0.737 |
|  | TI | 9.4 ± 0.6 | 9.4 ± 0.6 | -0.68 | 0.498 |
|  | TC | 9.4 ± 0.5 | 9.6 ± 0.4 | 5.97 | < 0.001 |
|  | MM | 9.4 ± 0.4 | 9.6 ± 0.3 | 3.92 | < 0.001 |
|  | LM | 11.5 ± 0.3 | 11.5 ± 0.3 | 0.69 | 0.488 |
| **3D Contrast** |  |  |  |  |  |
| (cm_contrast_3d_avg) |  |  |  |  |  |
|  | FE | 678.1 ± 89.9 | 685.1 ± 81.2 | 1.06 | 0.289 |
|  | FC | 394.6 ± 48.2 | 395.3 ± 49.5 | 0.20 | 0.842 |
|  | TI | 820.7 ± 185.7 | 843.3 ± 173.9 | 1.63 | 0.103 |
|  | TC | 773.1 ± 149.7 | 740.2 ± 129.5 | -3.05 | 0.002 |
|  | MM | 796.1 ± 127.1 | 768.4 ± 109.6 | -3.02 | 0.002 |
|  | LM | 299.8 ± 21.0 | 300.1 ± 20.6 | 0.22 | 0.825 |

Data are presented as mean ± standard deviation. Two-sided *P* values were calculated using two-sample independent t-tests. DMT, degenerative meniscal tear, FE: Femur, FC: Femoral Cartilage, TI: Tibia, TC: Tibial Cartilage, LM: Lateral Meniscus, MM: Medial Meniscus, 3D-DESS: 3-Dimensional Double-Echo Steady-State.

**Table S11. Longitudinal predictive discrimination and dynamic AUC across follow-up time horizons in the test cohort.**

| **Follow-up Time** | **Cases, n** | **Controls, n** | **MSC-RM AUC ^a^** | **CM AUC ^a^** | **ΔAUC ^b^** | ***P* Value ^c^** |
| --- | --- | --- | --- | --- | --- | --- |
| ≤1-Year (Rapid Onset) | 50 | 64 | 0.880 (0.814-0.938) | 0.466 (0.354-0.581) | 0.414 | < .001 |
| ≤2-Year | 58 | 64 | 0.867 (0.801-0.925) | 0.480 (0.375-0.589) | 0.387 | < .001 |
| ≤3-Year | 64 | 64 | 0.868 (0.803-0.924) | 0.501 (0.398-0.605) | 0.367 | < .001 |
| ≤4-Year (Overall Full Cohort) | 74 | 64 | 0.871 (0.802-0.918) | 0.517 (0.418-0.617) | 0.354 | < .001 |

^a^ 95% CI were empirically computed using 1,000 non-parametric bootstrap iterations.

^b^ Absolute increment in AUC achieved by the MSC-RM compared to the CM ($\Delta\text{AUC}=\text{AUC}_{\text{MSC-RM}}-\text{AUC}_{\text{CM}}$).

^c^ *P* values for comparative discrimination between MSC-RM and CM across each follow-up time horizon were evaluated using DeLong’s test for paired ROC curves. AUC: Area Under the receiver operating characteristic Curve, CI: Confidence Interval, CM: Clinical Model, DMT: Degenerative Meniscal Tear, MSC-RM: Multi-Structure and Clinical variable Radiomic Model.

**Table S12. Diagnostic performance and contingency classification of the MSC-RM under balanced versus high-specificity decision thresholds in the test cohort.**

| **Metric / Parameter** | **Balanced Strategy (Youden Index)** | **Conservative Screening Strategy (High Specificity ≥ 90%)** |
| --- | --- | --- |
| **Probability Cutoff Threshold** | 0.434 | 0.585 |
| **Diagnostic Performance (95% CI) ^a^** |  |  |
| Sensitivity (Recall), % | 71.9% (60.2%-81.9%) | 55.4% (43.4%-67.0%) |
| Specificity, % | 83.8% (72.9%-91.6%) | 98.4% (91.6%-99.9%) |
| PPV, % | 95.8% (85.7%-99.5%) | 97.6% (87.4%-99.9%) |
| NPV, % | 68.9% (58.3%-78.2%) | 65.6% (55.2%-75.0%) |
| PLR | 4.4 (2.56-7.7) | 35.5 (5.1-247.4) |
| NLR | 0.3 (0.2-0.5) | 0.5 (0.4-0.6) |
| DOR | 13.3 (5.6-31.4) | 78.3 (10.2-599.7) |
| Overall Accuracy, % | 78.3% (70.4%-84.9%) | 75.4% (67.3%-82.4%) |

^a^ Values are presented as percentage or ratio with 95% CI. The test cohort comprises n = 74 incident degenerative meniscal tear cases and n = 64 control knees followed over 4 years. 95% CIs for sensitivity, specificity, PPV, NPV, and accuracy were calculated using Clopper-Pearson exact binomial intervals; 95% CIs for PLR, NLR, and DOR were computed using log-transformed asymptotic standard errors. CI: Confidence Interval, DOR: Diagnostic Odds Ratio, MSC-RM: Multi-Structure and Clinical variable Radiomic Model, NLR: Negative Likelihood Ratio; NPV, Negative Predictive Value; PLR, Positive Likelihood Ratio; PPV: Positive Predictive Value.
